# Integrated genome-wide methylation and expression analyses reveal functional predictors of response to antidepressants

**DOI:** 10.1038/s41398-019-0589-0

**Published:** 2019-10-08

**Authors:** Chelsey Ju, Laura M. Fiori, Raoul Belzeaux, Jean-Francois Theroux, Gary Gang Chen, Zahia Aouabed, Pierre Blier, Faranak Farzan, Benicio N. Frey, Peter Giacobbe, Raymond W. Lam, Francesco Leri, Glenda M. MacQueen, Roumen Milev, Daniel J Müller, Sagar V. Parikh, Susan Rotzinger, Claudio N. Soares, Rudolf Uher, Qingqin Li, Jane A. Foster, Sidney H. Kennedy, Gustavo Turecki

**Affiliations:** 1 Department of Psychiatry, McGill Group for Suicide Studies, Douglas Mental Health University Institute, McGill University, Montreal, QC Canada; 2 Department of Psychiatry, Assistance Publique-Hopitaux de Marseille, Aix Marseille University, Marseille, France; 3 University of Ottawa Institute of Mental Health Research, Ottawa, K1Z 7K4 ON Canada; 4 Centre for Addiction and Mental Health, Toronto, ON M6J 1A8 Canada; 5 Mood Disorders Program, Department of Psychiatry and Behavioural Neurosciences, McMaster University; Women’s Health Concerns Clinic, St. Joseph’s Healthcare Hamilton, Hamilton, ON L8N 3K7 Canada; 6 Department of Psychiatry, University Health Network, University of Toronto, Toronto, ON M5T 2S8 Canada; 7 Department of Psychiatry, University of British Columbia, Vancouver, BC V6T 2A1 Canada; 8 Department of Psychology, University of Guelph, Guelph, ON N1G 2W1 Canada; 9 University of Calgary Hotchkiss Brain Institute, Calgary, AB T2N 1N4 Canada; 10 Providence Care Hospital, Kingston, ON K7L 4×3 Canada; 11 Department of Psychiatry, Queen’s University, Kingston, ON K7L 3N6 Canada; 12 University of Michigan, Ann Arbor, MI 48109 USA; 13 St Michael’s Hospital, Toronto, ON M5B 1M4 Canada; 14 MRC Social, Genetic and Developmental Psychiatry Centre, Institute of Psychiatry, Psychology & Neuroscience, King’s College London, London, SE5 8AF UK; 15 Department of Psychiatry, Dalhousie University, Halifax, NS B3H 2E2 Canada; 16 Janssen Research & Development, LLC, Pennington, NJ USA

**Keywords:** Depression, Predictive markers

## Abstract

Major depressive disorder (MDD) is primarily treated with antidepressants, yet many patients fail to respond adequately, and identifying antidepressant response biomarkers is thus of clinical significance. Some hypothesis-driven investigations of epigenetic markers for treatment response have been previously made, but genome-wide approaches remain unexplored. Healthy participants (*n* = 112) and MDD patients (*n* = 211) between 18–60 years old were recruited for an 8-week trial of escitalopram treatment. Responders and non-responders were identified using differential Montgomery-Åsberg Depression Rating Scale scores before and after treatment. Genome-wide DNA methylation and gene expression analyses were assessed using the Infinium MethylationEPIC Beadchip and HumanHT-12 v4 Expression Beadchip, respectively, on pre-treatment peripheral blood DNA and RNA samples. Differentially methylated positions (DMPs) located in regions of differentially expressed genes between responders (*n* = 82) and non-responders (*n* = 95) were identified, and technically validated using a targeted sequencing approach. Three DMPs located in the genes *CHN2* (cg23687322, *p* = 0.00043 and cg06926818, *p* = 0.0014) and *JAK2* (cg08339825, *p* = 0.00021) were the most significantly associated with mRNA expression changes and subsequently validated. Replication was then conducted with non-responders (*n* = 76) and responders (*n* = 71) in an external cohort that underwent a similar antidepressant trial. One *CHN2* site (cg06926818; *p* = 0.03) was successfully replicated. Our findings indicate that differential methylation at CpG sites upstream of the *CHN2* and *JAK2* TSS regions are possible peripheral predictors of antidepressant treatment response. Future studies can provide further insight on robustness of our candidate biomarkers, and greater characterization of functional components.

## Introduction

Antidepressants are considered an effective treatment option for major depressive disorder (MDD), a severe affective disorder that is currently deemed to be the leading cause of global disability^1^. However, treatment selection is clinically subjective, response is determined by trial and error, and objective patient improvement is difficult to distinguish from the placebo effect^2^. On average, 4 weeks are required for a notable response to treatment, and 6 weeks are required for symptom remission^3^. In addition to the long period of symptom evaluation, the uncomfortable side effects of antidepressants greatly contribute to noncompliance with treatment. Around 60% of patients fail to respond to initial interventions, whereas 20–30% of these patients do not respond despite multiple attempts^4,5^. Thus, a treatment paradigm that reliably matches patients with effective antidepressants as early on as possible would minimize their suffering, and avoid adversities associated with selecting appropriate medications. Predictive biomarkers for antidepressant response could greatly benefit clinical practice by decreasing the duration of evaluating drug efficacy^6^.

MDD is heterogeneous in symptom presentation and treatment response, and environmental factors have been shown to influence the onset, course and duration of illness^7^. Epigenetic modifiers of gene expression are key mediators of environmental effects on the genome. As such, exploring epigenetic mechanisms as possible predictors of treatment response is appealing, as they are better at reflecting the interaction of genetic and environmental factors. The most investigated and best characterized epigenetic modification in clinical studies is DNA methylation^8^. DNA methylation is defined by the addition of a methyl group typically to cytosine bases, and predominantly at those directly followed by a guanine (CpG dinucleotide sites). Differential methylation has been associated with life experiences such as drug abuse^9^, early childhood trauma^10^, and chronic stress^11^, all of which are predisposing factors for MDD.

DNA methylation-based biomarkers have already been successfully utilized for clinical evaluation of neurodevelopmental disorders^12^, multiple types of cancer^13^ and cardiovascular disease^14^. To date, only a few studies have investigated differential DNA methylation as a predictor biomarker at specific candidate loci that were previously associated with treatment response^15–17^, but no genome-wide study has been conducted.

Genome-wide analyses offer a non-biased experimental approach to identify novel candidates. To our knowledge, this is the first genome-wide differential DNA methylation conducted to identify possible predictors of antidepressant response. We compared responders (RES) and non-responders (NRES) to an eight-week escitalopram treatment. In addition, to investigate the possible functional role of identified methylation biomarkers, we also analyzed genome-wide differential gene expression. This information was used to select differentially methylated positions (DMPs) for further analyses.

## Materials and methods

### CAN-BIND-1 discovery cohort characterization

Our discovery cohort consisted of participants recruited for the Canadian Biomaker Integration Network in Depression (CAN-BIND-1), a multisite initiative dedicated to the discovery of treatment response biomarkers, which has been described in detail elsewhere^18^. Briefly, healthy control participants and MDD patients ranging between 18 and 60 years of age were enrolled in a prospective 16-week trial with escitalopram with an option of addition of aripiprazole at week 8. In this study, we are only analyzing baseline and week 8 data and outcomes. Healthy participants were included if they were free of psychiatric psychopathology and with no active medical diagnoses, and were matched for sex and age distribution with MDD patients. Depressed patients were excluded if they had other psychiatric diagnoses in addition to MDD, and if they had psychotic symptoms, high suicidality or concomitant neurological disorders, if they have already failed ≥4 pharmacological treatments for MDD, or previously failed to respond to escitalopram. Research Ethics Boards at all recruitment sites approved of the study design, and consent was obtained from all eligible participants for all procedures prior to the start of the trial. Following screening and recruitment, MDD diagnoses were clinically determined using the Mini International Neuropsychiatric Interview (MINI). All participants were assessed at baseline (W0) for symptom severity using the Montgomery Åsberg Depression Rating Scale (MADRS). MDD patients were administered escitalopram (10–20 mg/d) for 8 weeks. At week 8 (W8), MDD patients were assessed again with the MADRS. Escitalopram response was indicated by a ≥50% decrease in W8 MADRS scores relative to W0, and MDD patients were classified as either a responder (RES) or non-responder (NRES). Healthy controls underwent the same clinical assessments and evaluations as MDD patients at these time points, but did not receive any type of treatment or placebo. The CAN-BIND-1 clinical trial was registered with the ClinicalTrials.gov identification number: NCT01655706.

Demographic and clinical data were compared between HC, NRES and RES samples included in final analyses.

### Genome-wide DNA methylation analysis on the Infinium MethylationEPIC Beadchip

DNA was extracted from whole blood samples obtained from healthy controls and MDD patients at baseline prior to treatment, using a modified version of the Qiagen FlexiGene DNA kit. Bisulfite conversion, DNA quality control, genome-wide methylation analysis, and initial methylation signal detection quality control was performed at the McGill University and Genome Quebec Innovation Center (GQ). The Infinium MethylationEPIC Beadchip was used to assess genome-wide DNA methylation (Illumina, US). After accounting for attrition rates, and DNA sample quality control, pre-processing and analysis of raw microarray data for the remaining samples was conducted within R (ver 3.4) predominantly using the Chip Analysis Methylation Pipeline (ChAMP) Bioconductor package^19^, which utilizes many elements of minfi^20^. Sample methylation signal QC was assessed by plotting log median methylated and unmethylated signals. Samples were removed if they failed to cluster with others or if they exhibited lower median intensities in either signal channel. Probes with low signal detection relative to control probes, probes with <3 beads in >5% of samples, cross reactive probes, non-CpG probes, sex chromosome probes, and probes that hybridize to single nucleotide polymorphism sites were removed. Beta (β) values were calculated as the ratio of methylated signal to the sum of unmethylated and methylated signals at each CpG site, and subsequently normalized. log_2_ transformed β values were used for the remainder of pre-processing steps as recommended by Du et al.^21^, but reported as β values. Technical batches and covariates were detected using single value decomposition analysis. Detected and known batch effects were corrected for prior to differential methylation analysis. Differentially methylated positions (DMPs) were identified between NRES and RES using linear regression methods from the limma^22^, with age and sex as covariates. A 2% absolute change in average methylation (∆β) was set as a cutoff value to decrease the number of significant CpGs and identify sites with more biologically relevant methylation differences. A detailed account of our pre-processing and analysis steps for the MethylationEPIC Beadchip are included in Supplementary Methods.

### Genome-wide mRNA gene expression analysis on the HT-12 Beadchip

Baseline whole blood samples were obtained from HC and MDD participants in EDTA tubes containing LeukoLOCK filters (ThermoFisher, USA). Total RNA was extracted from filtered leukocytes using a modified version of the LeukoLOCK Total RNA Isolation System protocol, and treated with DNase to remove genomic DNA. RNA was converted to cRNA, and sent to GQ for further QC and analysis on HT-12 v4 Expression Beadchips (Illumina, USA). Pre-processing steps and differential gene expression analysis were performed in R using the *limma* Bioconductor package^22^. Only the subset of samples that appeared in our DNA methylation analysis were included. Probe signal detection, normalization, and filtering were conducted prior to differential expression analysis. Probes with a detection p-value <0.01 in at ≥20% of samples were retained. To assess differential expression, linear regression analyses on log_2_ transformed values was performed with age and sex accounted for as covariates. A detailed account of pre-processing and differential expression analysis is included in Supplementary Methods. Only probes with ≥±0.1 ∆logFC values between NRES and RES were included for further investigations. Genes that contained differentially methylated CpGs with average ∆β ≥ 2%, and that appeared in our differential expression analysis were identified.

### Targeted bisulfite sequencing for validation of genome-wide findings

Differentially methylated CpGs with an ∆β ≥ ±2% methylation and located in differentially expressed gene regions with a logFC ≥ 0.1 were selected for validation with targeted bisulfite sequencing on the Illumina MiSeq platform^23^. NRES and RES DNA samples were bisulfite converted using the Epitect 96 Bisulfite kit (Qiagen, USA) as per manufacturer’s guidelines. Primers were designed with the Methyl Primer Express software (ThermoFisher Scientific). All samples were ensured to have an optimal molarity of 2 nM prior to being loaded onto the MiSeq platform with the V3 600 cycle kit (Illumina, US). Methods. Specific details for primer design and amplicon library preparation are included in Supplementary Methods. Upon retrieving raw sequencing data, Trimmomatic (v.0.35) was used to trim adaptor sequences^24^. Reads with phred scores <20 were removed and aligned with Bowtie 2 (v 2.1.0)^25^. Methylated and non-methylated CpG signals were extracted to calculate the level of methylation at our sites of interest. Results were analyzed using one-tailed *t*-tests. Correlation of microarray and sequencing methylation values was assessed with Pearson correlation coefficients.

### Replication within the Douglas biomarker study

Replication was conducted using the Douglas Biomarker Study cohort, which was similarly designed to our discovery cohort. Participants were recruited at the Depressive Disorders Program at the Douglas Mental Health Institute, McGill University (Montreal, QC), and consisted of an 8-week antidepressant treatment for MDD patients randomly selected to receive either densvenlafaxine (serotonin and norepinephrine reuptake inhibitor; SNRI) or escitalopram (selective serotonin reuptake inhibitor; SSRI). Hamilton Depression Rating Scale (HAM-D) scores were used to assess symptom severity at baseline and W8, where a ≥ 50% relative decrease in HAM-D scores at W8 denoted a response. Genome-wide methylation analysis on the Infinium MethylationEPIC Beadchip was conducted at Illumina. Sample descriptive data were statistically analyzed similarly to what was described previously for our discovery cohort. We used the same pipeline described previously to analyze differential methylation, with additional covariate corrections made for antidepressant type. We compared differential methylation at our three probes of interest between MDD patients and psychiatrically healthy controls to ascertain whether our findings were specific to antidepressant response. After identifying our three CpGs of interest, we compared methylation level of healthy controls at those sites specifically to the methylation levels of non-responders and responders respectively using two-tailed *t*-tests accounting for equal variance.

### ROC curve analysis

Receiver-operating characteristic (ROC) curve analyses were performed to assess the ability of our successfully replicated CpG site cg06926818 to discriminate between non-responders and responders to antidepressant treatment. Discovery and replication cohort methylation levels at cg06926818 for responders and non-responders were utilized within SPSS to calculate sensitivity, specificity and confidence intervals of their respective ROC curves. Analysis of the ROC coordinates determined the area under the curve (AUC), which was used to assess prediction accuracy. AUC significance was determined using a *p*-value threshold of *p* < 0.05.

### Investigating effects of blood cell heterogeneity

Heterogeneity of white blood cell types has potential confounding effects on DNA methylation measurements based in peripheral blood samples^26^. To address the possibility of confounding effects of blood cell composition, complete blood cell counts were obtained from each patient during the trial. One-way ANOVA tests were used analyze all three comparison groups for any effects of blood cell proportions on our main results.

## Results

### CAN-BIND cohort characterization

**Fig. 1:**
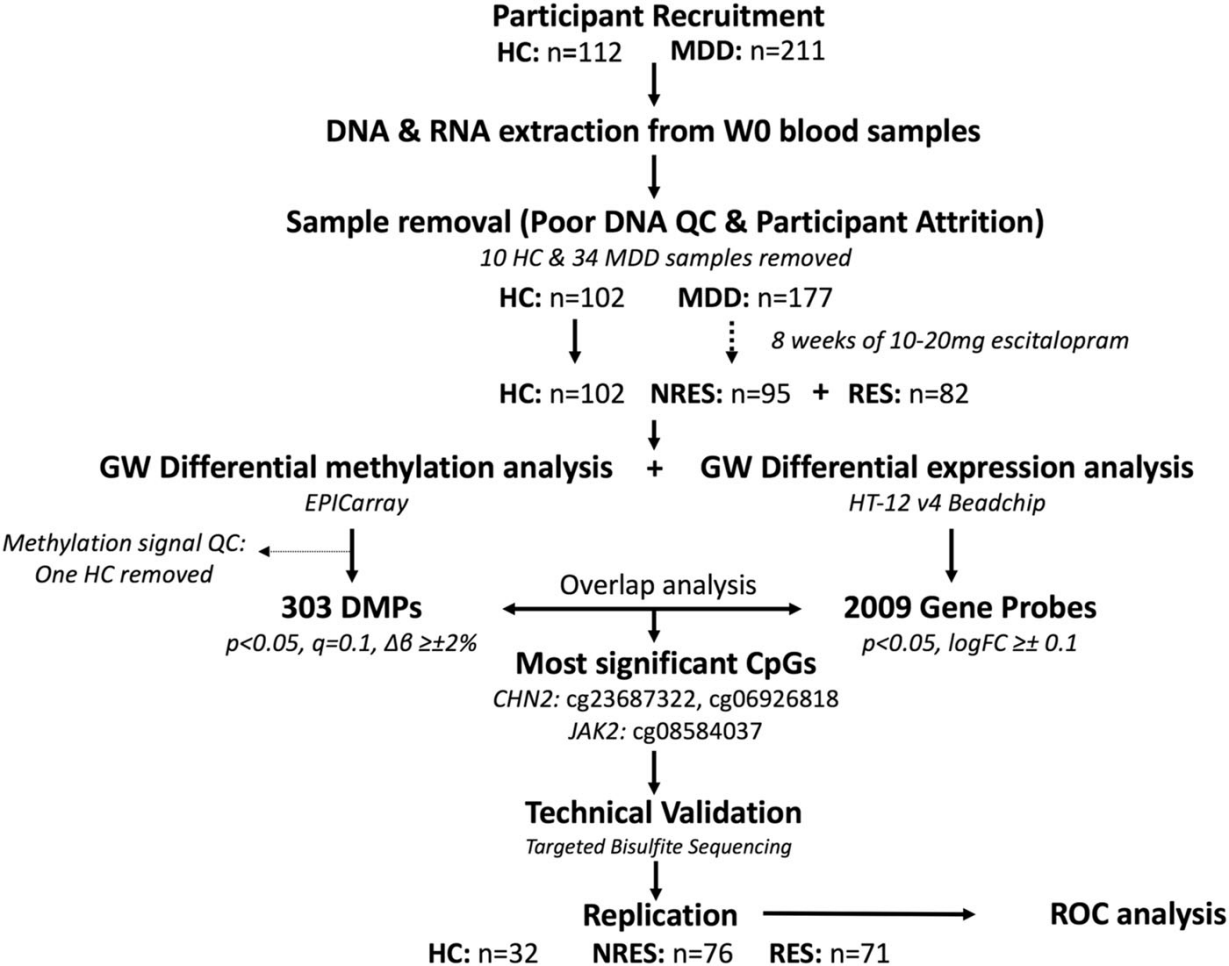
Process of research design

An overview of our research methodology is presented in Fig. 1. In our discovery cohort, 211 depressed patients and 112 healthy controls were initially recruited for the clinical trial. From these, 34 depressed patients and 10 healthy controls had to be excluded from further analyses because of unsuccessful completion of the trial, or poor DNA sample quality. One healthy control was removed due to poor methylation signal detection QC. Downstream analysis proceeded with 101 healthy control and 177 depressed subjects. Using differential MADRS scores, 95 NRES and 82 RES were identified within the MDD group.

For the remaining HC, NRES and RES samples, psychiatric and social demographics (including previous number of major depressive episodes, family history of psychiatric illnesses, age of MDD onset, highest level of education obtained, yearly income, marital status, and ethnicity) are provided in Table 1. No significant differences in age, gender, level of education, yearly income, marital status, ethnicity, number of previous major depressive episodes and age of MDD onset were noted between HC, NRES and RES groups. Significant differences were noted between all three groups for the following categories: MADRS scores at baseline (F = 1362.46, *p* < 0.05), MADRS scores at Week 8 (F = 466.17, *p* < 0.05) and family history of psychiatric illness (F = 36.04, *p* < 0.05). Post-hoc analyses revealed significant differences between HC and drug-treated groups for both baseline and post-treatment MADRS scores (*p* = 5.10E-9 for both comparisons). However, no significant differences between NRES and RES groups were noted for baseline MADRS scores (*p* = 0.181). Expectedly, we noted significant differences between NRES and RES post-treatment MADRS scores (*p* = 5.10E-9). There was a significant difference between HCs and drug-treated groups for a family history of psychiatric illness (*p* = 5.13E-9) but not between NRES and RES (*p* = 0.994).

**Table 1:** CAN-BIND-1 sample demographics

	HC	NRES	RES		Statistical analysis
*n*	101	95	82		
Gender—*n* (%)					F = 0.44, *p* = 0.64
Male	38 (37.6)	39 (41.1)	28 (34.1)		
Female	63 (62.4)	56 (58.9)	54 (65.9)		
Avg. Age	32.8	36	35		F = 1.85, *p* = 0.16
Std. Dev.	10.5	13.17	12.2		
Std. Error	1.04	1.35	1.35		
Avg. MADRS T0	0.9	30.5	29.3		F = 1362.46, *p* < 0.05^a^
Std. Dev	1.74	5.48	5.43	HC vs. NRES	*p* = 5.10E-9
Std. Error	0.17	0.58	0.61	HC vs. RES	*p* = 5.10E-9
				NRES vs. RES	*p* = 0.181
Avg. MADRS T8	1.1	23.9	7.9		F = 466.17, *p* < 0.05^a^
Std. Dev	2.20	7.30	4.99	HC vs. NRES	*p* = 5.10E-9
Std. Error	0.23	0.75	0.55	HC vs. RES	*p* = 5.10E-9
				NRES vs. RES	*p* = 5.10E-9
Education					F = 1.18, *p* = 0.31
High school	0	3	4		
High school diploma	11	16	12		
Bachelor’s	48	25	16		
Master’s	10	5	11		
PhD	7	0	0		
College (no degree)	8	28	18		
Associate degree	10	16	19		
Professional degree	7	1	0		
Prefer no answer	0	1	2		
Income					F = 1.47, *p* = 0.232
$0–24,999	14	19	18		
$25,000–50,000	15	17	21		
$50,000–75,000	22	20	10		
$75,000–99,999	13	9	9		
$>100,000	19	14	15		
Prefer No Answer	18	16	9		
Marital status					F = 0.98, *p* = 0.379
Never Married	55	49	50		
Married	39	29	21		
Divorced/Sep./Widowed	7	16	11		
Prefer no answer	0	1	0		
Ethnicity					F = 0.15, *p* = 0.864
Caucasian	70	66	58		
Black	3	4	3		
Hispanic	3	5	4		
Asian	18	12	11		
Other	4	4	6		
Prefer no answer	3	4	0		
Psych. family history					F = 36.04, *p* < 0.05^a^
Yes	21	66	57	HC vs. NRES	*p* = 5.13E-9
No	75	28	25	HC vs. RES	*p* = 5.13E-9
Prefer no answer	5	1	0	NRES vs. RES	*p* = 0.994
Previous MDE					*p* = 0.29
Yes (*n* = 1)	N/A	94	81		
No	N/A	0	1		
Prefer no Answer		1	0		
Avg. age of MDD onset	N/A	22	19.8		F = 1.88, *p* = 0.17
Std. dev	N/A	11.17	9.27		
Std. error	N/A	1.17	1.04		

Demographics for our discovery cohort. One-way ANOVA values comparing controls, non-responders and responders are displayed in the last column for all characteristics except for “Previous MDE”, where *t*-test results from comparing NRES and RES are displayed

*NRES* non-responder, *RES* responder, *MADRS* Montgomery Asberg Depression Rating Scale, *MDE* major depressive episode

^a^Tukey’s HSD post-hoc analysis results are also noted for characteristics with significant ANOVA results

### Differential methylation analysis

Pre-processing of raw data for retained samples was conducted within R using *ChAMP* and 679,362 CpG probes were retained for downstream analysis. We identified 2571 significantly DMPs (*p* < 0.05, *q* = 0.1); however, this included DMPs with very small differences in methylation (i.e. ∆β < 0.5%). Therefore, a ∆β ≥ ±2% cutoff was applied to identify 303 DMPs with methylation changes that are more likely to be biologically relevant (Supplementary Table 1).

### Differential mRNA expression analysis

Sixteen thousand three hundred seventy eight mRNA probes were retained and assessed for differential mRNA expression with linear regression analyses. A cutoff of logFC ≥ ±0.1 was used to eliminate gene probes with low levels of differential expression, resulting in 2009 retained probes. The remaining expression probes were overlapped with DMP probes with the intent to identify DMPs that are more likely to affect *cis* gene expression.

### CpG selection and validation

We overlapped the list of genes identified from our 303 significant DMPs with genes targeted by 2009 HT-12 probes to select DMPs for validation. Sixteen DMPs were located within genes that appeared on our list of 2009 expression probes (Supplementary Table 2), and all but two DMP probes overlapped with unique genes (Table 2). Of these 16 CpGs, *CHN2* and *JAK2* were the most significant differentially expressed genes after multiple testing corrections (*q* = 0.05). Thus, cg23687322 (*CHN2;* *p* = 1.93 × 10^−4^, *q* = 0.08, ∆β = −0.05), cg06926818 (*CHN2*; *p* = 9.67 × 10^−5^, *q* = 0.07, ∆β = −0.04) and cg08584037 (*JAK2;* *p* = 3.14 × 10^−4^, *q* = 0.09, ∆β = −0.02) were selected for targeted validation. All three CpG probes were located within 1500 bp of the TSS of their respective genes, and responders were observed to have relative decreased methylation compared to non-responders.

**Table 2:** Sixteen gene probes identified from differential expression analysis that contained significant DMPs with Δ β ≥ ±2%

Probe_ID	Gene	AveExpr	t	*p*.val	FDR	logFC
ILMN_1772540	ATMIN	5.694	−2.338	0.020	0.08	−0.105
ILMN_2223720	ATMIN	7.142	1.749	0.081	0.11	0.150
ILMN_1730291	ATP1B1	5.207	2.141	0.033	0.08	0.113
ILMN_3244172	CD52	11.269	−2.231	0.027	0.08	−0.161
ILMN_2403237	CHN2	6.391	−2.775	0.006	0.05	−0.139
ILMN_1774110	CHN2	5.326	−2.094	0.037	0.08	−0.112
ILMN_2140799	FAM24B	5.469	−2.113	0.036	0.08	−0.109
ILMN_1728799	FBP1	7.394	−1.747	0.082	0.11	−0.107
ILMN_3246953	FTSJD2	6.601	−2.046	0.042	0.08	−0.101
ILMN_1683178	JAK2	7.110	−2.754	0.006	0.05	−0.126
ILMN_1695812	KRT72	5.715	−0.988	0.324	0.34	−0.137
ILMN_2216815	MAP7	5.656	1.718	0.087	0.11	0.127
ILMN_2075794	NLRP8	11.263	1.478	0.141	0.16	0.205
ILMN_1737252	NRG1	5.664	−1.476	0.141	0.16	−0.169
ILMN_1693341	SNRPN	7.368	1.935	0.054	0.09	0.122
ILMN_1671442	WDR43	5.423	−2.380	0.018	0.08	−0.102

Fourteen unique genes overlapped between our differential methylation and differential expression analyses. Fold change (FC) is in reference to RES

**Fig. 2:**
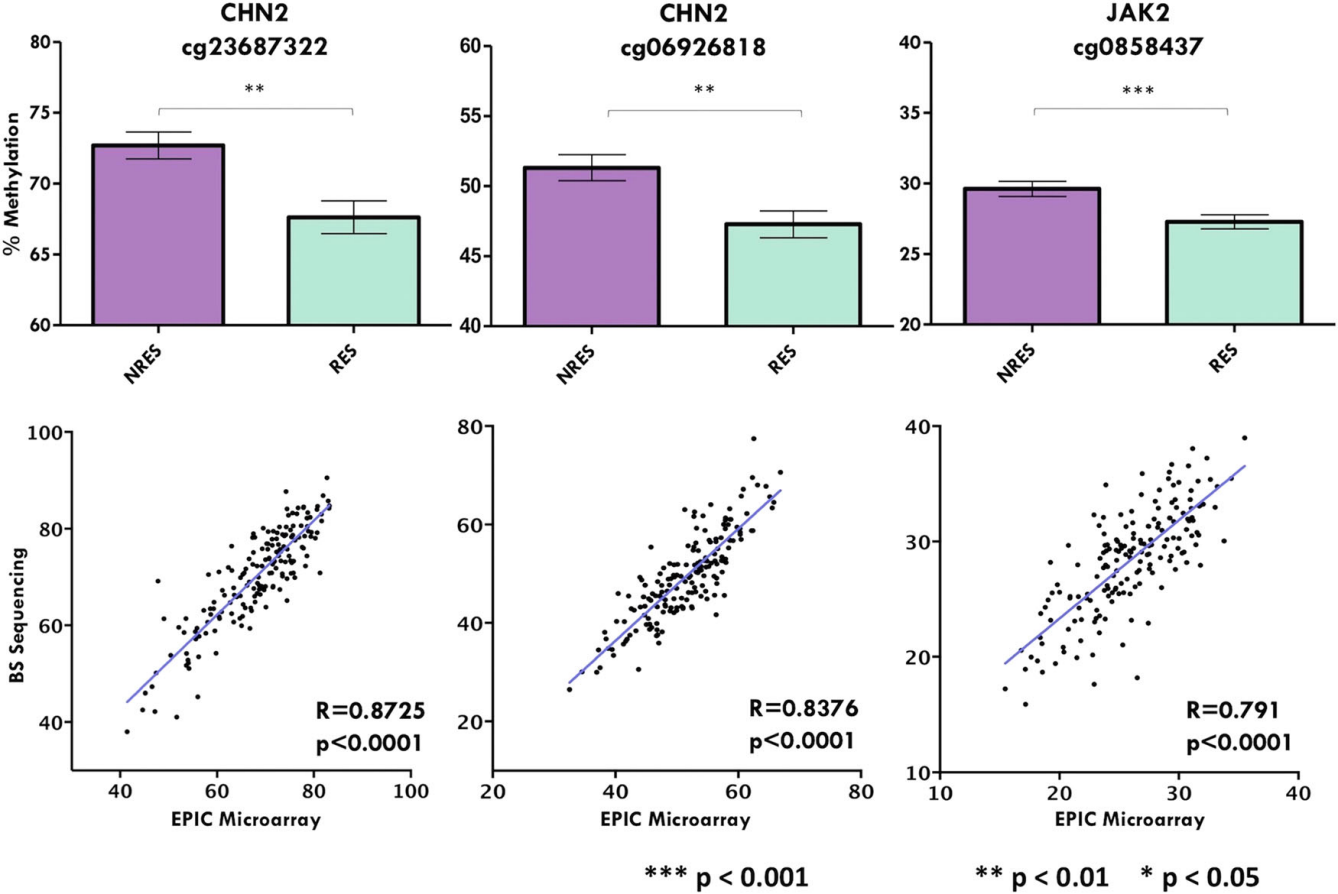
Differential Methylation between NRES and RES at cg23687322, cg06926818 and cg0858437 assessed through EPICarray analysis, correlated with values assessed through targeted bisulfite sequencing. Bar graphs show % methylation of NRES and RES detected through targeting bisulfite sequencing methods at cg23687322 (*p* = 0.0009), cg06926818 (*p* = 0.0058) and cg08584037 (*p* = 0.0009). Scatterplots show correlation of methylation levels assessed by EPIC microarray and targeted bisulfite sequencing platforms for cg23687322, cg08339825 and cg08584037. R Pearson correlation coefficient

Validation was conducted with 92 NRES and 83 RES samples. Targeted bisulfite sequencing of CpG probes within *CHN2 (*cg23687322, *p* = 0.0016 and cg06926818, *p* = 0.0058) and *JAK2* (cg08584037, *p* = 0.0009) (Fig. 2a–c and Supplementary Table 3). The level of CpG methylation assessed by targeted bisulfite sequencing and microarray methods were significantly correlated (*p* < 0.0001) with relatively high Pearson correlation coefficients for all three CpG probes (cg23687322, *r* = 0.87; cg06926818, *r* = 0.84; cg08584037, *r* = 0.72; Fig. 2d–f).

### Blood cell heterogeneity

The individual proportion of lymphocytes, monocytes, neutrophils, eosinophils and basophils did not have any specific, significant effects on our primary findings (Supplementary Table 4).

### Replication in the Douglas biomarker study cohort

Thirty one HC, 76 NRES, and 71 RES samples from the Douglas Biomarker Study cohort were used for replicating our methylation findings at cg23687322, cg06926818 and cg08584037 between RES and NRES. Detailed sample characterization of the replication cohort is provided in Table 3.

**Table 3:** Douglas biomarker study replication cohort characteristics

	HC	NRES	RES	Statistical analysis	
*n*	31	76	71		
Gender—*n* (%)					F = 0.78, *p* = 0.47
Male	15	27	28		
Female	16 (52)	49 (64.5)	43 (60.6)		
Age	47	41	39.2		F = 4.75, *p* < 0.05^a^
Std. Dev.	14.13	12.56	11.68	HC vs. NRES	*p* = 0.07
Std. Error	2.54	1.44	1.39	HC vs. RES	*p* = 0.01
				NRES vs. RES	*p* = 0.50
HAM-D T0	0.75	33.5	31.3		F = 321.59, *p* < 0.05^a^
Std. Dev.	1.00	6.33	6.89	HC vs. NRES	*p* = 5.10 E-9
Std. error	0.19	0.73	0.82	HC vs. RES	*p* = 5.10 E-9
				NRES vs. RES	*p* = 0.07
HAM-D T8	1.6	25.2	8.6		F = 206.46, *p* < 0.05^a^
Std. Dev.	2.00	7.51	5.67	HC vs. NRES	*p* = 5.10 E-9
Std. error	0.38	0.86	0.67	HC vs. RES	*p* = 3.00 E-6
				NRES vs. RES	*p* = 5.10 E-9
Medication					F = 0.31, *p* = 0.58
Cipralex/SSRI	N/A	35	36		
Pristiq/SNRI	N/A	41	35		
Ethnicity					F = 2.78, *p* = 0.07
Caucasian	25	63	54		
Black	0	1	1		
Hispanic	1	3	3		
Asian	1	4	4		
Other	2	2	9		
Prefer no answer	2	3	0		

Demographics for our replication cohort. One-way ANOVA values comparing controls, non-responders and responders are displayed in the last column for all characteristics

*HC* healthy controls, *NRES* non-responders, *RES* responders, *HAM-D*  Hamilton Depression Scale

^a^Tukey’s post-HOC analysis results are displayed below significant ANOVA values

In *CHN2* gene regions, differential methylation at cg06926818 (*p* = 0.027, ∆β = −0.03) was successfully replicated. There was no significant difference in methylation at cg06926818 when comparing healthy controls to NRES (*p* = 0.74) and to RES (*p* = 0.21). Although of similar magnitude and direction, differential methylation at cg23687322 did not reach significance in this cohort (*p* = 0.17, ∆β = −0.03). We did not replicate the cg08584037 position in *JAK2* (*p* = 0.59, ∆β = −0.003).

### ROC curve analysis

**Fig. 3:**
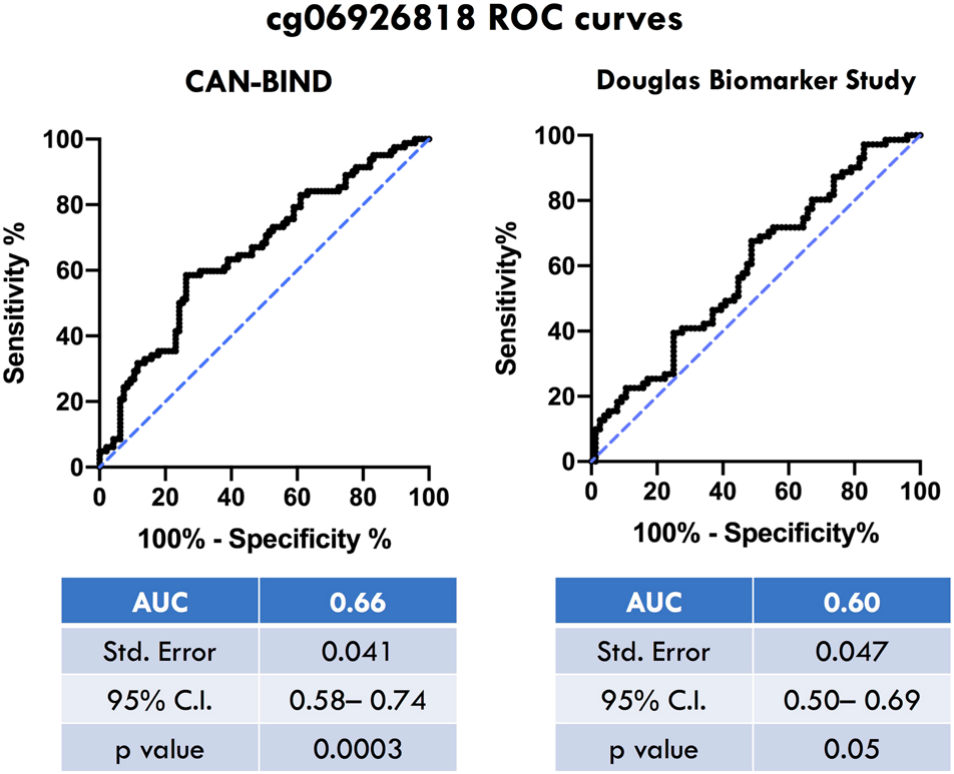
ROC curves for cg06926818. ROC curves were derived from methylation values of our discovery cohort (left) and replication cohort (right). AUC area under the curve, C.I. confidence interval

ROC analysis with AUC calculations for cg06926818 between non-responders and responders were used to assess their potential predictive value as biomarkers in both discovery and replication cohorts (Fig. 3). The AUC for the ROC curve of cg06926818 was 0.66 in our discovery cohort (*p* = 0.0003, C.I. = 0.58–0.74) and 0.59 in our replication cohort (*p* = 0.05, C.I. = 0.50–0.69). The ROC curves for cg23687322 and cg08584037 are displayed in Supplementary Fig. 1.

## Discussion

This study represents the first genome-wide differential methylation analysis of antidepressant response in clinically depressed patients. We identified significantly differentially methylated CpGs associated with antidepressant response through a genome-wide method in peripheral blood samples retrieved prior to receiving 10–20 mg of escitalopram treatment. We applied a stringent ∆β cutoff and incorporated associated differential gene expression data in order select DMPs with a functional component. This allowed us to identify cg23687322, cg06926818 and cg06926818 located in *CHN2* and *JAK2* gene regions as our candidate predictors of response.

Differential methylation levels at all three of our CpGs of interest were relatively low. This presents the question of whether there was a true distinction between comparison groups. However, small differential values are commonly reported in studies of psychiatric disorders^27–29^ and these subtle differences are thought to reflect the complex etiology and course of multifactorial illnesses such as MDD. For psychiatric diseases overall, biomarkers will likely be established by collectively considering a panel of multiple differential findings rather than through the standard method of identifying high fold changes of one specific observation. Additionally, many other social and lifestyle factors, such as dietary habits^30^, smoking history^31^ and chronic stress^32^, are shown to have specific effects on the methylome landscape, and were not accounted for as covariates in our analysis. We were ultimately able to validate and correlate our microarray-based findings through targeted bisulfite sequencing, and replicated differential methylation at cg06926818. Future genome-wide methylation studies will provide additional information on the robustness of our biomarker candidates.

All three CpGs of interest were located in promoter regions of their respective genes, specifically within 1500 bp upstream of the transcription start site. Increased DNA methylation at CpGs in promoter regions, in particular, is usually associated with a decrease in mRNA expression. However, our data showed that responders exhibited relative decreases in both DNA methylation and mRNA expression at all three CpG sites compared to non-responders. Possible explanations for these findings include the action of other regulatory elements, such as enhancers and DNA methyl-transferases, which may be exerting additional secondary effects on gene expression and methylation. In addition, 3D chromatin structure may have regulatory effects on gene expression, particularly through distant, trans-acting mechanisms. For example, insulators can prevent an enhancer from activating a promoter through long-range interactions with other regulator elements^33^. Finally, the combined expression of multiple loci on separate chromosomes, and their subsequent interactions can also activate or repress opposing epigenetic states^34^. Overall, the complex regulation between methylation and expression implied through our findings likely reflects the intricate relationship between predisposing genetic factors and environmental effects on MDD etiology, symptom severity and variation in treatment response.

Differential *CHN2* methylation has not been directly assessed in the context of treatment response or MDD, but it has is implicated in disorders that often co-occur with MDD or depressive symptoms, such as substance abuse^35^, ADHD^36^ and psychosis^37^. *CHN2*, or β2-chimaerin, maps to chromosome 7p15.3 and encodes for a GTP-ase activating protein predominantly expressed in the pancreas and brain^37^. In the brain, *CHN2* observed to have a role in neurodevelopmental hippocampal axon pruning. From animal-based studies, adult hippocampal neurogenesis has been observed to be stimulated by antidepressant administration^38,39^, and additionally shown to be a prerequisite for a behavioural response to all major antidepressants^40^. Thus, it is possible that differential baseline methylation levels at cg23687322 and cg06926818 within *CHN2* could therefore reflect the epigenetic regulation of certain molecular processes in the brain, such as hippocampal neurogenesis, that are required for eliciting antidepressant response.

*JAK2*, or Janus kinase 2, maps to chromosome 9p24.1 and encodes for an intracellular, non-receptor tyrosine kinase. Upon activation of JAK tyrosine kinase activity, a family of transcription factors called STATs (signal transducer and activators of transcription) are further activated to initiate downstream regulatory activity^41^. Similarly, to *CHN2*, differential methylation at *JAK2* may also have functional regulatory effects on hippocampal neurogenesis that could be associated with antidepressant response. Ketamine, an acutely acting antidepressant, reverses stress-induced learning deficits in adult rats and increases *Arc* levels (a synaptic plasticity consolidating protein) only in the presence of *JAK2*. Furthermore, phosphorylated JAK2 colocalizes with Arc in dendritic spines, showing evident JAK2/STAT signaling during synaptic plasticity events^42^. However, *JAK2* also has important functions in an inflammation, which is important to consider in the context of depression and treatment response. It has a non-redundant role in cytokine receptor signaling pathways, which mediate components of innate and adaptive immunity^43^. Increased peripheral inflammation has been associated with poorer response to antidepressant medication^44^, and inflammatory components associated with JAK/STAT signaling^43^ (i.e. interleukin-6*; IL-6* and C-reactive protein; *CRP*) have been as treatment response predictor biomarkers^45^. Differential methylation in *JAK2* is possibly a secondary indicator to the underlying variation of patient inflammation levels that predict response. Moreover, the use of SSRI or SNRI antidepressants are also associated with changes in levels of inflammation^46^. Serotonin and norepinephrine moderate differential cytokine production, and a chronic imbalance between the two can modify the ratio of cytokine types^47^. These differential effects of neurotransmitter levels on inflammation may be related to why our *JAK2* probe was not indicative of treatment response in the replication cohort given that it involved both SSRI and SNRI treatment. Differential methylation at cg08584037 in *JAK2* is potentially only predictive of SSRI response, but not SNRI response due to the effects of neurotransmitter imbalance on cytokine production. Although we did correct for antidepressant type as a covariate, it would be interesting to note whether differential methylation at cg08584037 in *JAK2* would be replicated in future studies that specific to SSRI treatment.

There are a number of specific limitations that should be considered in this current study. Firstly, by applying stringent differential methylation and expression cutoffs, this excludes many potentially interesting sites for evaluation. Although differential cutoff values are valid approaches for site selection in genome-wide approaches, they do not take into account the subtle genetic changes that are likely reflected by the heterogeneity of psychiatric disease phenotypes. Secondly, the EPICarray targets specific CpGs and non-CpGs sites, and thus, other genomic methylation sites that could play a role in antidepressant response may not have been investigated. Thirdly, the technical methods used for differential methylation analyses do not distinguish between hydroxymethylated cytosines and methylated cytosines. Hydroxymethlyated cytosines are often found in gene bodies, and all three CpGs of interest were located in gene promoter regions^48^, decreasing the likelihood that our main findings are affected by this limitation. Further, our studies were conducted in peripheral blood samples^49–51^, which may not represent methylation processes in the brain, the target organ of depression.

Finally, the strength of our findings demonstrated through replication and ROC curve analysis indicate that it may not be feasible clinically to rely on methylation at one CpG site alone as a predictive biomarker. This is unsurprising, given the complex nature of psychiatric illness, and the multitude of underlying genetic and environmental factors that may contribute to manifestation and the course of disease. Thus, our results promote the concept of multiple biomarkers (or “biosignatures”) being used together, although our results also suggest that gene expression may be a more powerful biomarker than methylation.

This study is the first to conduct genome-wide differential DNA methylation analysis associated with antidepressant response from peripheral blood DNA samples of MDD patients. Three DMPs were identified, and technically validated using targeted bisulfite sequencing. One CpG site within *CHN2* was further replicated in an independent cohort. Overall, our findings provide initial evidence for the role of epigenetic factors in treatment, and propose new predictors of antidepressant response. Future studies, using larger sample sizes or longitudinal designs with multiple timepoints should be conducted in order to increase power of antidepressant biomarker studies. Robustness is the most important clinical consideration for biomarkers, and as more genome-wide investigations are conducted across independent cohorts, this will provide future opportunities for further replication and clinical consideration (especially when considered alongside other predictive biomarkers) for our proposed predictors of treatment response.

## Supplementary information

Supplementary Materials

Supplementary Figure 1

Supplementary Figure 1 Legend
