# Supplementary material for: Integrated genome-wide methylation and expression analyses reveal functional predictors of response to antidepressants
Source: Transl Psychiatry. 2019 Oct 8;9:254. doi: 10.1038/s41398-019-0589-0 (PMC6783543; doi:10.1038/s41398-019-0589-0)
Supplement: Supplementary file 1 — Supplementary Materials [file 41398_2019_589_MOESM1_ESM.docx]

**SUPPLEMENTARY MATERIALS**

**TABLES**

**Supplementary Table 1: 303 DMPs in genomic regions with at least ±2% Δ** **β values.**

|  | **Gene** | **chr** | **bp** | **feature** | **cgi** | **p val** | **FDR** | **NRES** | **RES** | **Δbeta** |
| --- | --- | --- | --- | --- | --- | --- | --- | --- | --- | --- |
| cg11249728 | ACSL3 | 2 | 223800611 | Body | opensea | 2.04E-04 | 8.43E-02 | 0.608 | 0.584 | -0.024 |
| cg03261737 | ADGRG1 | 16 | 57666593 | 5'UTR | opensea | 3.97E-05 | 5.19E-02 | 0.581 | 0.557 | -0.024 |
| cg10772169 | AGL | 1 | 100315213 | TSS1500 | shore | 1.62E-06 | 2.27E-02 | 0.527 | 0.505 | -0.021 |
| cg15720535 | AGPAT2 | 9 | 139582585 | TSS1500 | island | 2.35E-04 | 8.77E-02 | 0.529 | 0.486 | -0.043 |
| cg12461099 | ALDH4A1 | 1 | 19217794 | TSS1500 | opensea | 1.82E-04 | 8.16E-02 | 0.578 | 0.558 | -0.021 |
| cg14344550 | ALK | 2 | 29516596 | Body | opensea | 8.81E-06 | 3.65E-02 | 0.199 | 0.241 | 0.042 |
| cg08166588 | AMIGO3 | 3 | 49757438 | TSS1500 | shore | 7.00E-07 | 1.93E-02 | 0.627 | 0.599 | -0.028 |
| cg15486224 | ANKRD46 | 8 | 101527561 | Body | opensea | 1.93E-06 | 2.39E-02 | 0.385 | 0.326 | -0.059 |
| cg03497652 | ANKS3 | 16 | 4751569 | Body | opensea | 1.40E-04 | 7.44E-02 | 0.597 | 0.574 | -0.022 |
| cg09950162 | ANO4 | 12 | 101480520 | Body | opensea | 9.05E-06 | 3.65E-02 | 0.594 | 0.559 | -0.035 |
| cg07737560 | ANO4 | 12 | 101470827 | Body | opensea | 1.85E-04 | 8.18E-02 | 0.500 | 0.530 | 0.030 |
| cg16362232 | ANO9 | 11 | 430036 | Body | shore | 3.27E-04 | 9.58E-02 | 0.755 | 0.728 | -0.028 |
| cg12655260 | ARHGAP26 | 5 | 142562569 | Body | opensea | 1.25E-04 | 7.25E-02 | 0.361 | 0.304 | -0.057 |
| cg17140497 | ARHGAP26 | 5 | 142563177 | Body | opensea | 8.53E-05 | 6.42E-02 | 0.210 | 0.186 | -0.024 |
| cg11710969 | ARMC3 | 10 | 23217173 | 5'UTR | opensea | 1.90E-04 | 8.28E-02 | 0.280 | 0.256 | -0.024 |
| cg03493768 | ART3 | 4 | 76996690 | 5'UTR | opensea | 1.06E-04 | 6.80E-02 | 0.645 | 0.620 | -0.025 |
| cg09868768 | ASPRV1 | 2 | 70188605 | 1stExon | island | 2.33E-04 | 8.75E-02 | 0.630 | 0.609 | -0.021 |
| cg25337691 | ATG9B | 7 | 150717629 | Body | shore | 3.19E-04 | 9.51E-02 | 0.581 | 0.561 | -0.020 |
| cg02745111 | ATMIN | 16 | 81070647 | Body | shore | 9.60E-05 | 6.62E-02 | 0.351 | 0.328 | -0.023 |
| cg20182111 | ATP13A4 | 3 | 193217253 | Body | opensea | 3.01E-04 | 9.35E-02 | 0.615 | 0.570 | -0.044 |
| cg13104274 | ATP1B1 | 1 | 169078316 | Body | shore | 4.77E-05 | 5.56E-02 | 0.790 | 0.770 | -0.020 |
| cg26009832 | ATP1B1 | 1 | 169081894 | Body | opensea | 9.50E-05 | 6.62E-02 | 0.673 | 0.653 | -0.020 |
| cg21187669 | ATPAF2 | 17 | 17929033 | Body | opensea | 2.61E-04 | 8.98E-02 | 0.573 | 0.551 | -0.022 |
| cg00195322 | B4GALT5 | 20 | 48278559 | Body | opensea | 1.06E-04 | 6.80E-02 | 0.425 | 0.401 | -0.024 |
| cg15812976 | BAG1 | 9 | 33256910 | ExonBnd | opensea | 4.85E-05 | 5.58E-02 | 0.748 | 0.725 | -0.023 |
| cg08500171 | BAT2 | 6 | 31590674 | Body | shore | 3.78E-05 | 5.16E-02 | 0.705 | 0.685 | -0.021 |
| cg23202887 | BCL11B | 14 | 99691372 | Body | opensea | 2.16E-04 | 8.54E-02 | 0.507 | 0.529 | 0.022 |
| cg11519176 | BGLAP | 1 | 156211801 | TSS200 | shelf | 3.35E-04 | 9.65E-02 | 0.393 | 0.368 | -0.025 |
| cg19749188 | BICD1 | 12 | 32351549 | Body | opensea | 1.00E-04 | 6.72E-02 | 0.809 | 0.788 | -0.021 |
| cg16053902 | BRD1 | 22 | 50181906 | Body | shelf | 3.44E-04 | 9.75E-02 | 0.809 | 0.774 | -0.035 |
| cg12096447 | BRF1 | 14 | 105714400 | TSS200 | shore | 1.48E-04 | 7.68E-02 | 0.690 | 0.713 | 0.023 |
| cg17226676 | BSPRY | 9 | 116126574 | Body | opensea | 1.34E-04 | 7.33E-02 | 0.534 | 0.501 | -0.032 |
| cg18039797 | BUB1B | 15 | 40509518 | Body | opensea | 8.43E-06 | 3.65E-02 | 0.491 | 0.464 | -0.027 |
| cg08021797 | C16orf74 | 16 | 85785130 | TSS1500 | island | 6.72E-05 | 6.02E-02 | 0.481 | 0.459 | -0.022 |
| cg24766229 | C17orf99 | 17 | 76151124 | Body | opensea | 3.57E-04 | 9.87E-02 | 0.400 | 0.378 | -0.022 |
| cg17234962 | C1R | 12 | 7241782 | Body | opensea | 5.68E-06 | 3.54E-02 | 0.630 | 0.605 | -0.025 |
| cg07959070 | C22orf34 | 22 | 50026188 | Body | island | 7.62E-06 | 3.65E-02 | 0.775 | 0.685 | -0.090 |
| cg18527739 | C22orf34 | 22 | 49946312 | Body | opensea | 8.34E-06 | 3.65E-02 | 0.820 | 0.761 | -0.059 |
| cg04138436 | C22orf34 | 22 | 49822980 | Body | opensea | 2.76E-04 | 9.12E-02 | 0.729 | 0.692 | -0.038 |
| cg03814063 | C22orf34 | 22 | 50025915 | Body | island | 3.30E-05 | 4.93E-02 | 0.816 | 0.785 | -0.031 |
| cg00783170 | C22orf34 | 22 | 49861705 | Body | opensea | 1.73E-05 | 4.12E-02 | 0.885 | 0.855 | -0.030 |
| cg06355422 | C22orf34 | 22 | 50013649 | Body | shelf | 7.06E-05 | 6.12E-02 | 0.844 | 0.816 | -0.028 |
| cg01681680 | C22orf34 | 22 | 49855255 | Body | opensea | 8.60E-06 | 3.65E-02 | 0.884 | 0.859 | -0.025 |
| cg15823183 | C3orf56 | 3 | 126911942 | TSS200 | opensea | 1.31E-04 | 7.31E-02 | 0.843 | 0.865 | 0.022 |
| cg24642844 | C7orf50 | 7 | 1081250 | Body | shore | 2.57E-04 | 8.94E-02 | 0.799 | 0.766 | -0.033 |
| cg02210115 | CAB39L | 13 | 49990767 | 5'UTR | opensea | 3.57E-05 | 5.11E-02 | 0.617 | 0.592 | -0.025 |
| cg06831653 | CACNA1E | 1 | 181748219 | Body | opensea | 1.05E-04 | 6.80E-02 | 0.819 | 0.798 | -0.022 |
| cg13532410 | CACNA2D3 | 3 | 54732582 | Body | opensea | 1.81E-04 | 8.15E-02 | 0.745 | 0.768 | 0.023 |
| cg20106684 | CACNG3 | 16 | 24269504 | Body | shore | 1.67E-04 | 7.99E-02 | 0.603 | 0.636 | 0.033 |
| cg22536580 | CALCB | 11 | 15095822 | 5'UTR | island | 2.98E-05 | 4.76E-02 | 0.165 | 0.139 | -0.025 |
| cg14551034 | CAPRIN1 | 11 | 34094566 | Body | opensea | 4.54E-06 | 3.37E-02 | 0.484 | 0.453 | -0.030 |
| cg21646082 | CCDC21 | 1 | 26603970 | 3'UTR | shelf | 5.28E-07 | 1.93E-02 | 0.810 | 0.790 | -0.020 |
| cg25215890 | CD48 | 1 | 160651452 | Body | opensea | 3.73E-04 | 9.99E-02 | 0.474 | 0.451 | -0.023 |
| cg07060948 | CD48 | 1 | 160651479 | Body | opensea | 1.98E-04 | 8.36E-02 | 0.211 | 0.191 | -0.020 |
| cg19677267 | CD52 | 1 | 26645161 | Body | opensea | 2.10E-04 | 8.49E-02 | 0.253 | 0.288 | 0.035 |
| cg12001491 | CD52 | 1 | 26645487 | Body | opensea | 3.50E-04 | 9.80E-02 | 0.356 | 0.396 | 0.040 |
| cg26117104 | CDK5RAP1 | 20 | 31975140 | ExonBnd | opensea | 4.40E-06 | 3.37E-02 | 0.821 | 0.781 | -0.040 |
| cg23687322 | CHN2 | 7 | 29523056 | TSS1500 | opensea | 1.93E-04 | 8.28E-02 | 0.715 | 0.667 | -0.048 |
| cg06926818 | CHN2 | 7 | 29523160 | TSS1500 | opensea | 9.67E-05 | 6.62E-02 | 0.537 | 0.499 | -0.038 |
| cg11803859 | CHST15 | 10 | 125770124 | Body | opensea | 2.33E-04 | 8.75E-02 | 0.558 | 0.530 | -0.028 |
| cg19236247 | CIB4 | 2 | 26835353 | Body | opensea | 9.68E-06 | 3.65E-02 | 0.482 | 0.434 | -0.049 |
| cg04471485 | CLIC3 | 9 | 139889789 | Body | island | 8.08E-05 | 6.36E-02 | 0.549 | 0.526 | -0.024 |
| cg06682371 | CMTM8 | 3 | 32291082 | Body | opensea | 3.10E-04 | 9.42E-02 | 0.269 | 0.243 | -0.025 |
| cg04269043 | CNGB1 | 16 | 57918043 | 3'UTR | island | 2.04E-04 | 8.43E-02 | 0.302 | 0.279 | -0.023 |
| cg13904574 | CNTFR | 9 | 34586050 | 5'UTR | shelf | 8.40E-06 | 3.65E-02 | 0.227 | 0.250 | 0.024 |
| cg17444747 | COL23A1 | 5 | 177915909 | Body | opensea | 2.51E-04 | 8.88E-02 | 0.516 | 0.480 | -0.035 |
| cg21871330 | COL23A1 | 5 | 177882221 | Body | opensea | 2.17E-04 | 8.56E-02 | 0.775 | 0.754 | -0.021 |
| cg09001527 | COL23A1 | 5 | 177942856 | Body | shelf | 2.04E-04 | 8.43E-02 | 0.764 | 0.743 | -0.021 |
| cg07427642 | COL23A1 | 5 | 177944298 | Body | shore | 2.03E-04 | 8.42E-02 | 0.583 | 0.562 | -0.021 |
| cg15301006 | CPLX2 | 5 | 175267609 | 5'UTR | opensea | 8.85E-05 | 6.48E-02 | 0.611 | 0.651 | 0.040 |
| cg19421526 | CRTAC1 | 10 | 99734513 | Body | opensea | 1.79E-05 | 4.18E-02 | 0.235 | 0.199 | -0.036 |
| cg25758699 | CRYGS | 3 | 186257301 | Body | opensea | 1.12E-04 | 6.94E-02 | 0.633 | 0.608 | -0.025 |
| cg05492904 | CYP19A1 | 15 | 51604503 | 5'UTR | opensea | 2.80E-04 | 9.14E-02 | 0.497 | 0.468 | -0.030 |
| cg16170087 | CYP1B1-AS1 | 2 | 38368819 | Body | opensea | 6.22E-05 | 5.94E-02 | 0.480 | 0.439 | -0.041 |
| cg18148659 | DENND4A | 15 | 65953468 | 3'UTR | opensea | 3.58E-04 | 9.88E-02 | 0.679 | 0.659 | -0.020 |
| cg07373298 | DISC1FP1 | 11 | 90434175 | Body | opensea | 3.19E-04 | 9.51E-02 | 0.859 | 0.839 | -0.020 |
| cg02209770 | DLGAP4 | 20 | 35062903 | Body | shore | 3.18E-04 | 9.51E-02 | 0.504 | 0.482 | -0.022 |
| cg20109856 | DLX6AS | 7 | 96643454 | TSS200 | shelf | 1.66E-06 | 2.27E-02 | 0.469 | 0.430 | -0.039 |
| cg08180998 | DNAJC15 | 13 | 43663213 | Body | opensea | 6.23E-06 | 3.54E-02 | 0.734 | 0.712 | -0.022 |
| cg27209571 | DNER | 2 | 230563222 | Body | opensea | 1.66E-04 | 7.99E-02 | 0.691 | 0.712 | 0.021 |
| cg08549898 | DOCK8 | 9 | 296755 | Body | opensea | 1.79E-04 | 8.13E-02 | 0.703 | 0.678 | -0.024 |
| cg22340526 | DPP6 | 7 | 153586207 | Body | shore | 1.05E-04 | 6.80E-02 | 0.781 | 0.760 | -0.020 |
| cg21989229 | DSE | 6 | 116608164 | 5'UTR | opensea | 1.02E-04 | 6.77E-02 | 0.605 | 0.575 | -0.030 |
| cg25700077 | DTX2P1-UPK3BP1-PMS2P11 | 7 | 76637488 | Body | opensea | 3.32E-05 | 4.94E-02 | 0.777 | 0.744 | -0.034 |
| cg26739697 | DTX2P1-UPK3BP1-PMS2P11 | 7 | 76637493 | Body | opensea | 1.13E-05 | 3.67E-02 | 0.848 | 0.820 | -0.029 |
| cg19689387 | EIF5 | 14 | 103807182 | Body | opensea | 3.02E-04 | 9.35E-02 | 0.495 | 0.468 | -0.027 |
| cg18892446 | ENC1 | 5 | 73938574 | TSS1500 | shore | 3.72E-04 | 9.99E-02 | 0.561 | 0.534 | -0.026 |
| cg24343097 | ENTPD5 | 14 | 74486664 | TSS1500 | shore | 6.67E-05 | 6.00E-02 | 0.416 | 0.395 | -0.021 |
| cg03502625 | EPB41 | 1 | 29212825 | TSS1500 | shore | 2.15E-04 | 8.52E-02 | 0.568 | 0.544 | -0.024 |
| cg22056241 | EPHA1 | 7 | 143107282 | TSS1500 | opensea | 5.42E-05 | 5.72E-02 | 0.701 | 0.679 | -0.022 |
| cg07068406 | EPHB1 | 3 | 134647778 | Body | opensea | 5.99E-05 | 5.86E-02 | 0.507 | 0.387 | -0.119 |
| cg10785929 | EPHB1 | 3 | 134650410 | Body | opensea | 8.90E-05 | 6.50E-02 | 0.817 | 0.739 | -0.078 |
| cg04425710 | ESCO2 | 8 | 27630920 | TSS1500 | shore | 2.26E-04 | 8.65E-02 | 0.693 | 0.717 | 0.023 |
| cg21025681 | EXOSC10 | 1 | 11134131 | Body | opensea | 9.71E-06 | 3.65E-02 | 0.689 | 0.666 | -0.023 |
| cg06279274 | FAM24B | 10 | 124635805 | 5'UTR | shelf | 1.31E-04 | 7.31E-02 | 0.739 | 0.713 | -0.026 |
| cg15355800 | FAM45A | 10 | 120895864 | Body | opensea | 2.55E-05 | 4.59E-02 | 0.615 | 0.583 | -0.033 |
| cg24508168 | FAM83F | 22 | 40405832 | Body | opensea | 2.35E-05 | 4.56E-02 | 0.262 | 0.293 | 0.031 |
| cg06705237 | FBP1 | 9 | 97402555 | TSS200 | shore | 2.47E-04 | 8.87E-02 | 0.561 | 0.537 | -0.024 |
| cg15621260 | FIBIN | 11 | 27015813 | 5'UTR | opensea | 4.79E-05 | 5.57E-02 | 0.278 | 0.245 | -0.034 |
| cg00700214 | FMO2 | 1 | 171154802 | 5'UTR | opensea | 1.43E-04 | 7.55E-02 | 0.727 | 0.702 | -0.025 |
| cg14422240 | FTSJD2 | 6 | 37425031 | Body | opensea | 7.06E-05 | 6.12E-02 | 0.690 | 0.661 | -0.028 |
| cg03149245 | GALNT9 | 12 | 132703598 | Body | opensea | 3.67E-05 | 5.14E-02 | 0.301 | 0.329 | 0.028 |
| cg05521767 | GDPD5 | 11 | 75230135 | 5'UTR | opensea | 1.43E-04 | 7.56E-02 | 0.505 | 0.485 | -0.020 |
| cg07805029 | GFI1 | 1 | 92953256 | TSS1500 | shore | 2.98E-04 | 9.33E-02 | 0.607 | 0.580 | -0.028 |
| cg26393275 | GPD2 | 2 | 157435476 | Body | opensea | 9.36E-05 | 6.60E-02 | 0.601 | 0.579 | -0.022 |
| cg00081729 | GREM2 | 1 | 240656737 | Body | shore | 2.51E-04 | 8.88E-02 | 0.698 | 0.663 | -0.035 |
| cg20716668 | GRK5 | 10 | 121043687 | Body | opensea | 3.31E-04 | 9.60E-02 | 0.543 | 0.566 | 0.023 |
| cg14479617 | GSK3B | 3 | 119542274 | 3'UTR | opensea | 1.80E-04 | 8.15E-02 | 0.736 | 0.714 | -0.023 |
| cg25210835 | GSTM5 | 1 | 110254828 | TSS200 | opensea | 1.78E-04 | 8.13E-02 | 0.321 | 0.241 | -0.080 |
| cg24467349 | GSTM5 | 1 | 110254835 | TSS200 | opensea | 2.15E-04 | 8.52E-02 | 0.353 | 0.277 | -0.076 |
| cg14377951 | GSTM5 | 1 | 110254896 | 1stExon | opensea | 2.47E-04 | 8.87E-02 | 0.350 | 0.287 | -0.063 |
| cg25535106 | GTF3C2 | 2 | 27549046 | 3'UTR | opensea | 3.15E-05 | 4.82E-02 | 0.687 | 0.666 | -0.020 |
| cg11987759 | GUSB | 7 | 65425863 | 3'UTR | opensea | 9.68E-05 | 6.62E-02 | 0.847 | 0.816 | -0.031 |
| cg26214742 | H2AFY | 5 | 134735914 | TSS1500 | shore | 2.84E-05 | 4.71E-02 | 0.436 | 0.410 | -0.026 |
| cg00057840 | HDAC4 | 2 | 240076109 | Body | opensea | 2.27E-04 | 8.69E-02 | 0.461 | 0.435 | -0.026 |
| cg18564053 | HES3 | 1 | 6303793 | TSS1500 | shore | 1.10E-04 | 6.91E-02 | 0.201 | 0.177 | -0.023 |
| cg21718051 | HIVEP1 | 6 | 12071462 | Body | opensea | 2.94E-04 | 9.30E-02 | 0.565 | 0.540 | -0.026 |
| cg02549973 | HMHA1 | 19 | 1076202 | TSS1500 | shore | 2.56E-05 | 4.59E-02 | 0.699 | 0.725 | 0.026 |
| cg24987751 | HPSE2 | 10 | 100276194 | Body | opensea | 5.94E-05 | 5.86E-02 | 0.664 | 0.684 | 0.020 |
| cg20464360 | HSF5 | 17 | 56564855 | Body | island | 1.39E-05 | 3.88E-02 | 0.512 | 0.541 | 0.029 |
| cg22922494 | IL12A-AS1 | 3 | 159647642 | Body | opensea | 1.81E-04 | 8.15E-02 | 0.323 | 0.296 | -0.026 |
| cg21593409 | IL17C | 16 | 88706389 | Body | island | 2.50E-04 | 8.88E-02 | 0.507 | 0.479 | -0.028 |
| cg07794885 | IL17C | 16 | 88703611 | TSS1500 | shelf | 1.40E-04 | 7.44E-02 | 0.540 | 0.512 | -0.028 |
| cg10479672 | IL1F8 | 2 | 113810641 | TSS1500 | shore | 3.91E-05 | 5.19E-02 | 0.694 | 0.718 | 0.023 |
| cg00756845 | IPCEF1 | 6 | 154678593 | TSS1500 | opensea | 9.31E-05 | 6.59E-02 | 0.653 | 0.623 | -0.030 |
| cg03615426 | IQCK | 16 | 19777410 | Body | opensea | 2.75E-04 | 9.11E-02 | 0.304 | 0.396 | 0.092 |
| cg21951975 | IRF6 | 1 | 209979733 | TSS1500 | shore | 6.08E-05 | 5.87E-02 | 0.182 | 0.144 | -0.038 |
| cg25192855 | IRF6 | 1 | 209979283 | 5'UTR | shore | 8.52E-05 | 6.42E-02 | 0.228 | 0.207 | -0.021 |
| cg16134369 | IRX4 | 5 | 1888009 | TSS1500 | shore | 2.52E-04 | 8.88E-02 | 0.367 | 0.347 | -0.020 |
| cg02287260 | ITPK1 | 14 | 93510671 | Body | opensea | 1.79E-04 | 8.14E-02 | 0.745 | 0.721 | -0.023 |
| cg12534855 | ITPR1 | 3 | 4735740 | Body | opensea | 2.42E-04 | 8.85E-02 | 0.626 | 0.595 | -0.030 |
| cg06287611 | ITPR2 | 12 | 26624359 | Body | opensea | 3.22E-04 | 9.52E-02 | 0.424 | 0.399 | -0.025 |
| cg08584037 | JAK2 | 9 | 4984071 | TSS1500 | shore | 3.14E-04 | 9.45E-02 | 0.268 | 0.247 | -0.021 |
| cg10975897 | JARID2 | 6 | 15504844 | Body | opensea | 3.63E-04 | 9.93E-02 | 0.515 | 0.493 | -0.022 |
| cg11738976 | JPH2 | 20 | 42744590 | Body | island | 8.13E-06 | 3.65E-02 | 0.345 | 0.320 | -0.025 |
| cg17238677 | KCNN3 | 1 | 154736223 | Body | shelf | 9.56E-08 | 9.37E-03 | 0.756 | 0.735 | -0.021 |
| cg20204316 | KCNQ3 | 8 | 133460603 | TSS1500 | opensea | 1.32E-04 | 7.31E-02 | 0.633 | 0.612 | -0.021 |
| cg00399027 | KIAA0182 | 16 | 85676861 | 5'UTR | shore | 2.10E-04 | 8.49E-02 | 0.500 | 0.479 | -0.022 |
| cg03182584 | KIAA0895 | 7 | 36364854 | 3'UTR | opensea | 1.10E-04 | 6.91E-02 | 0.600 | 0.578 | -0.022 |
| cg02881189 | KIAA1539 | 9 | 35111032 | 5'UTR | shore | 4.69E-05 | 5.56E-02 | 0.462 | 0.437 | -0.024 |
| cg00620464 | KIAA1715 | 2 | 176868389 | TSS1500 | shore | 4.94E-05 | 5.60E-02 | 0.726 | 0.698 | -0.028 |
| cg07149296 | KIAA1755 | 20 | 36889389 | TSS1500 | island | 2.32E-04 | 8.75E-02 | 0.366 | 0.340 | -0.026 |
| cg26856575 | KIF1B | 1 | 10291784 | 5'UTR | opensea | 1.63E-05 | 4.04E-02 | 0.681 | 0.660 | -0.020 |
| cg04382643 | KLC3 | 19 | 45849853 | Body | island | 2.21E-05 | 4.49E-02 | 0.555 | 0.515 | -0.040 |
| cg26299044 | KRT12 | 17 | 39021588 | Body | shore | 4.61E-05 | 5.56E-02 | 0.568 | 0.538 | -0.030 |
| cg22200736 | KRT72 | 12 | 52995358 | TSS200 | shore | 3.20E-04 | 9.51E-02 | 0.428 | 0.406 | -0.022 |
| cg12693179 | LGR5 | 12 | 71863439 | Body | opensea | 1.49E-05 | 3.94E-02 | 0.738 | 0.716 | -0.022 |
| cg12398777 | LINC00968 | 8 | 57472469 | TSS200 | opensea | 2.13E-04 | 8.52E-02 | 0.327 | 0.300 | -0.027 |
| cg14684596 | LINC01268 | 6 | 114191904 | Body | opensea | 3.67E-04 | 9.95E-02 | 0.759 | 0.735 | -0.024 |
| cg08153693 | LINC01289 | 8 | 64680636 | TSS1500 | opensea | 8.49E-05 | 6.42E-02 | 0.529 | 0.552 | 0.023 |
| cg00117532 | LINGO1 | 15 | 78098084 | 5'UTR | opensea | 3.02E-04 | 9.35E-02 | 0.368 | 0.345 | -0.023 |
| cg09754549 | LOC100130274 | 8 | 144790656 | TSS1500 | island | 1.02E-04 | 6.77E-02 | 0.766 | 0.795 | 0.030 |
| cg03431084 | LOC100131496 | 20 | 45948853 | Body | opensea | 1.93E-04 | 8.29E-02 | 0.632 | 0.610 | -0.021 |
| cg02272576 | LOC100132354 | 6 | 43868964 | Body | opensea | 8.39E-06 | 3.65E-02 | 0.611 | 0.561 | -0.050 |
| cg08627981 | LOC100289473 | 20 | 1757237 | Body | shore | 5.57E-05 | 5.74E-02 | 0.219 | 0.185 | -0.034 |
| cg18270009 | LOC100506869 | 12 | 59198886 | Body | opensea | 3.58E-04 | 9.87E-02 | 0.329 | 0.356 | 0.028 |
| cg12234768 | LOC101928371 | 2 | 88862824 | Body | opensea | 3.20E-04 | 9.51E-02 | 0.681 | 0.653 | -0.028 |
| cg00967229 | LOC101928978 | 4 | 85180506 | Body | opensea | 2.55E-04 | 8.92E-02 | 0.388 | 0.367 | -0.021 |
| cg06686396 | LOC101928989 | 11 | 82026140 | Body | opensea | 8.77E-05 | 6.48E-02 | 0.315 | 0.351 | 0.036 |
| cg18950481 | LOC149134 | 1 | 246952889 | TSS200 | shore | 4.77E-05 | 5.56E-02 | 0.825 | 0.805 | -0.020 |
| cg17429662 | LOC149373 | 1 | 231323501 | TSS200 | opensea | 1.40E-05 | 3.88E-02 | 0.568 | 0.599 | 0.031 |
| cg24587835 | LOC339166 | 17 | 5674234 | TSS1500 | opensea | 4.29E-06 | 3.37E-02 | 0.391 | 0.523 | 0.132 |
| cg15252215 | LOC399815 | 10 | 124639110 | TSS200 | island | 1.58E-04 | 7.88E-02 | 0.205 | 0.230 | 0.026 |
| cg11216554 | LOC399815 | 10 | 124638983 | TSS200 | island | 1.61E-04 | 7.96E-02 | 0.414 | 0.451 | 0.037 |
| cg01091620 | LOH12CR1 | 12 | 12561471 | Body | opensea | 2.85E-04 | 9.18E-02 | 0.235 | 0.211 | -0.024 |
| cg24845165 | LPIN3 | 20 | 39968231 | TSS1500 | shore | 7.24E-05 | 6.16E-02 | 0.630 | 0.607 | -0.023 |
| cg13816228 | LRRC8B | 1 | 90022780 | TSS1500 | opensea | 1.67E-04 | 7.99E-02 | 0.530 | 0.482 | -0.048 |
| cg09272338 | LRRC8B | 1 | 90013382 | 5'UTR | opensea | 2.12E-05 | 4.43E-02 | 0.661 | 0.636 | -0.024 |
| cg15791719 | LRRC8D | 1 | 90354310 | 5'UTR | opensea | 1.14E-04 | 7.01E-02 | 0.659 | 0.630 | -0.029 |
| cg05542101 | MACC1 | 7 | 20186624 | Body | opensea | 3.08E-05 | 4.79E-02 | 0.622 | 0.594 | -0.028 |
| cg10153341 | MAN1A1 | 6 | 119665694 | Body | shelf | 3.85E-05 | 5.19E-02 | 0.664 | 0.632 | -0.032 |
| cg13950452 | MAP1B | 5 | 71463696 | Body | opensea | 2.18E-05 | 4.49E-02 | 0.769 | 0.748 | -0.021 |
| cg04453169 | MAP7 | 6 | 136680760 | Body | shore | 3.94E-05 | 5.19E-02 | 0.501 | 0.466 | -0.036 |
| cg21099759 | MARCH7 | 2 | 160567993 | TSS1500 | shore | 2.39E-04 | 8.80E-02 | 0.568 | 0.545 | -0.024 |
| cg10639811 | MBNL1 | 3 | 152083133 | Body | opensea | 2.95E-04 | 9.32E-02 | 0.578 | 0.553 | -0.025 |
| cg20553766 | MC3R | 20 | 54824583 | 1stExon | island | 1.35E-05 | 3.88E-02 | 0.448 | 0.493 | 0.045 |
| cg06269415 | MCC | 5 | 112602827 | Body | opensea | 6.69E-05 | 6.01E-02 | 0.460 | 0.440 | -0.021 |
| cg10230190 | MCOLN2 | 1 | 85405081 | Body | opensea | 3.52E-04 | 9.81E-02 | 0.553 | 0.524 | -0.029 |
| cg22676212 | MEIS3 | 19 | 47910108 | Body | island | 6.27E-05 | 5.94E-02 | 0.554 | 0.513 | -0.041 |
| cg19273694 | MFSD2B | 2 | 24233923 | Body | shore | 1.58E-05 | 3.99E-02 | 0.731 | 0.708 | -0.023 |
| cg21356710 | MFSD2B | 2 | 24234017 | Body | shore | 2.26E-04 | 8.65E-02 | 0.552 | 0.530 | -0.023 |
| cg00784161 | MLPH | 2 | 238406432 | Body | opensea | 6.30E-05 | 5.94E-02 | 0.802 | 0.781 | -0.022 |
| cg09805466 | MOGAT1 | 2 | 223566483 | Body | opensea | 2.07E-04 | 8.46E-02 | 0.433 | 0.457 | 0.024 |
| cg08663592 | MUC16 | 19 | 8989084 | Body | opensea | 2.65E-04 | 9.01E-02 | 0.421 | 0.446 | 0.025 |
| cg09271052 | MUC22 | 6 | 30977529 | 5'UTR | opensea | 4.65E-05 | 5.56E-02 | 0.759 | 0.783 | 0.025 |
| cg23738210 | MYO18B | 22 | 26253182 | Body | opensea | 2.69E-04 | 9.05E-02 | 0.570 | 0.592 | 0.023 |
| cg09269848 | MYO1C | 17 | 1396074 | TSS200 | shore | 1.89E-04 | 8.24E-02 | 0.342 | 0.314 | -0.028 |
| cg22795769 | MYO1C | 17 | 1396123 | TSS200 | shore | 1.32E-04 | 7.31E-02 | 0.754 | 0.733 | -0.021 |
| cg15699693 | MYOZ3 | 5 | 150054944 | Body | shelf | 2.99E-04 | 9.33E-02 | 0.549 | 0.517 | -0.032 |
| cg25457884 | MYT1 | 20 | 62796136 | 5'UTR | opensea | 1.83E-04 | 8.16E-02 | 0.785 | 0.810 | 0.025 |
| cg04287574 | NAV1 | 1 | 201619622 | Body | island | 3.27E-04 | 9.58E-02 | 0.343 | 0.288 | -0.055 |
| cg19095920 | NBLA00301 | 4 | 174458819 | Body | shore | 1.75E-04 | 8.08E-02 | 0.217 | 0.192 | -0.025 |
| cg12778228 | NCRNA00188 | 17 | 16341601 | TSS1500 | shore | 1.38E-04 | 7.42E-02 | 0.549 | 0.525 | -0.023 |
| cg19282259 | NCRNA00200 | 10 | 1205611 | TSS200 | island | 2.85E-04 | 9.18E-02 | 0.865 | 0.839 | -0.025 |
| cg21773245 | NDNF | 4 | 121983226 | 5'UTR | opensea | 3.15E-04 | 9.48E-02 | 0.571 | 0.543 | -0.028 |
| cg07623113 | NDUFB10 | 16 | 2008723 | TSS1500 | shore | 5.49E-05 | 5.74E-02 | 0.720 | 0.686 | -0.033 |
| cg18581616 | NLRP8 | 19 | 56478019 | Body | opensea | 4.25E-06 | 3.37E-02 | 0.816 | 0.845 | 0.029 |
| cg18496287 | NRD1 | 1 | 52259574 | Body | opensea | 3.81E-05 | 5.18E-02 | 0.548 | 0.525 | -0.023 |
| cg03485252 | NRG1 | 8 | 31503975 | Body | opensea | 1.05E-05 | 3.67E-02 | 0.721 | 0.744 | 0.023 |
| cg13610659 | OBFC2B | 12 | 56622608 | Body | opensea | 4.95E-05 | 5.60E-02 | 0.663 | 0.638 | -0.025 |
| cg11359094 | OPRD1 | 1 | 29172578 | Body | opensea | 1.05E-05 | 3.67E-02 | 0.604 | 0.576 | -0.028 |
| cg17212470 | OR1J4 | 9 | 125280584 | TSS1500 | opensea | 1.23E-04 | 7.25E-02 | 0.577 | 0.603 | 0.026 |
| cg20576955 | OR52I2 | 11 | 4606701 | TSS1500 | opensea | 5.96E-06 | 3.54E-02 | 0.785 | 0.807 | 0.022 |
| cg13816428 | OR5AU1 | 14 | 21625173 | TSS1500 | opensea | 1.33E-04 | 7.31E-02 | 0.502 | 0.534 | 0.032 |
| cg11320244 | OSBPL1A | 18 | 21795073 | Body | opensea | 3.58E-04 | 9.87E-02 | 0.622 | 0.589 | -0.032 |
| cg21449673 | PAAF1 | 11 | 73618548 | Body | opensea | 2.65E-04 | 9.01E-02 | 0.344 | 0.302 | -0.042 |
| cg12575659 | PACRG-AS1 | 6 | 163746319 | TSS1500 | opensea | 5.65E-05 | 5.79E-02 | 0.229 | 0.206 | -0.023 |
| cg27000690 | PACSIN1 | 6 | 34437227 | 5'UTR | shelf | 1.11E-04 | 6.93E-02 | 0.624 | 0.601 | -0.022 |
| cg24025782 | PACSIN2 | 22 | 43327600 | 5'UTR | opensea | 3.21E-04 | 9.52E-02 | 0.307 | 0.279 | -0.028 |
| cg14353649 | PAOX | 10 | 135191496 | TSS1500 | shore | 2.85E-04 | 9.18E-02 | 0.459 | 0.438 | -0.021 |
| cg09685257 | PARD3 | 10 | 34686368 | Body | opensea | 3.72E-04 | 9.99E-02 | 0.619 | 0.597 | -0.021 |
| cg04276953 | PAX7 | 1 | 18980700 | Body | opensea | 1.27E-04 | 7.28E-02 | 0.450 | 0.424 | -0.026 |
| cg12080079 | PAX7 | 1 | 19007925 | Body | opensea | 5.23E-07 | 1.93E-02 | 0.742 | 0.783 | 0.040 |
| cg21805940 | PCCA | 13 | 101174420 | Body | opensea | 3.43E-04 | 9.74E-02 | 0.465 | 0.436 | -0.029 |
| cg12526318 | PCDHB17 | 5 | 140535392 | TSS200 | shore | 2.64E-04 | 9.00E-02 | 0.186 | 0.208 | 0.022 |
| cg16179521 | PCSK6 | 15 | 102009894 | Body | opensea | 3.47E-04 | 9.77E-02 | 0.289 | 0.265 | -0.024 |
| cg11229771 | PDE6B | 4 | 640503 | Body | opensea | 9.47E-05 | 6.62E-02 | 0.736 | 0.766 | 0.030 |
| cg26693817 | PDE6B | 4 | 640348 | Body | opensea | 6.00E-05 | 5.86E-02 | 0.662 | 0.705 | 0.042 |
| cg23954416 | PDPN | 1 | 13909161 | TSS1500 | shore | 4.51E-07 | 1.93E-02 | 0.778 | 0.740 | -0.038 |
| cg06452518 | PEPD | 19 | 33923307 | Body | opensea | 1.53E-04 | 7.75E-02 | 0.496 | 0.470 | -0.025 |
| cg00846554 | PHACTR1 | 6 | 12748001 | Body | shore | 3.62E-05 | 5.12E-02 | 0.436 | 0.413 | -0.023 |
| cg00275896 | PHACTR3 | 20 | 58251756 | 1stExon | opensea | 3.49E-05 | 5.05E-02 | 0.704 | 0.734 | 0.030 |
| cg05688588 | PI4K2B | 4 | 25237268 | Body | shore | 7.29E-05 | 6.18E-02 | 0.304 | 0.342 | 0.038 |
| cg17865045 | PILRA | 7 | 99994933 | Body | opensea | 1.25E-05 | 3.80E-02 | 0.440 | 0.408 | -0.032 |
| cg18319102 | PIWIL1 | 12 | 130822256 | TSS200 | shore | 1.32E-05 | 3.86E-02 | 0.422 | 0.357 | -0.065 |
| cg24838063 | PIWIL1 | 12 | 130822603 | TSS200 | island | 2.71E-04 | 9.06E-02 | 0.742 | 0.681 | -0.061 |
| cg24229701 | PIWIL1 | 12 | 130821962 | TSS1500 | shore | 1.43E-04 | 7.56E-02 | 0.627 | 0.576 | -0.052 |
| cg09858226 | PKNOX1 | 21 | 44401549 | 5'UTR | opensea | 1.27E-05 | 3.80E-02 | 0.800 | 0.778 | -0.023 |
| cg08385266 | PLB1 | 2 | 28769125 | Body | opensea | 5.33E-05 | 5.69E-02 | 0.475 | 0.442 | -0.033 |
| cg22412747 | PLB1 | 2 | 28768014 | Body | opensea | 2.44E-05 | 4.57E-02 | 0.343 | 0.311 | -0.032 |
| cg22784187 | PON3 | 7 | 95025407 | Body | shore | 2.02E-04 | 8.41E-02 | 0.496 | 0.472 | -0.024 |
| cg25844590 | PPFIBP2 | 11 | 7621556 | Body | opensea | 3.28E-04 | 9.58E-02 | 0.566 | 0.530 | -0.036 |
| cg03186149 | PPP5C | 19 | 46877200 | Body | opensea | 1.58E-04 | 7.88E-02 | 0.593 | 0.568 | -0.025 |
| cg05257528 | PRTN3 | 19 | 846179 | Body | island | 1.83E-04 | 8.16E-02 | 0.581 | 0.557 | -0.024 |
| cg17144383 | PTPDC1 | 9 | 96868748 | Body | opensea | 8.35E-05 | 6.42E-02 | 0.721 | 0.699 | -0.023 |
| cg11055991 | PTPRN2 | 7 | 158280410 | Body | shore | 1.59E-04 | 7.90E-02 | 0.619 | 0.591 | -0.028 |
| cg09865698 | PVRL1 | 11 | 119597471 | Body | shore | 2.94E-05 | 4.74E-02 | 0.516 | 0.496 | -0.021 |
| cg21602651 | RAB3GAP2 | 1 | 220397618 | Body | opensea | 6.63E-05 | 6.00E-02 | 0.466 | 0.434 | -0.032 |
| cg00227342 | RAB5C | 17 | 40284353 | 5'UTR | opensea | 5.60E-06 | 3.54E-02 | 0.382 | 0.354 | -0.028 |
| cg15975554 | RABGAP1L | 1 | 174959074 | Body | opensea | 5.39E-06 | 3.49E-02 | 0.739 | 0.713 | -0.026 |
| cg13580105 | RAP1GAP | 1 | 21975062 | Body | shelf | 1.13E-04 | 6.94E-02 | 0.609 | 0.574 | -0.035 |
| cg04062715 | RASGEF1C | 5 | 179632567 | 5'UTR | shelf | 2.70E-05 | 4.68E-02 | 0.698 | 0.678 | -0.020 |
| cg00009085 | RBM33 | 7 | 155473359 | Body | opensea | 5.74E-06 | 3.54E-02 | 0.633 | 0.611 | -0.022 |
| cg10527482 | RELL1 | 4 | 37676204 | Body | opensea | 2.57E-04 | 8.94E-02 | 0.672 | 0.646 | -0.026 |
| cg08047233 | RERE | 1 | 8578167 | Body | opensea | 2.15E-04 | 8.52E-02 | 0.652 | 0.629 | -0.023 |
| cg11505048 | RGL1 | 1 | 183622726 | 5'UTR | opensea | 3.27E-04 | 9.58E-02 | 0.632 | 0.611 | -0.021 |
| cg11283152 | RHOBTB1 | 10 | 62749211 | Body | opensea | 1.03E-04 | 6.77E-02 | 0.761 | 0.704 | -0.057 |
| cg10180052 | RNF151 | 16 | 2018558 | Body | island | 1.24E-04 | 7.25E-02 | 0.708 | 0.680 | -0.028 |
| cg16738194 | RNF212 | 4 | 1076636 | Body | opensea | 2.03E-06 | 2.39E-02 | 0.579 | 0.559 | -0.020 |
| cg20980321 | RNF5 | 6 | 32144667 | TSS1500 | opensea | 2.77E-04 | 9.12E-02 | 0.448 | 0.425 | -0.024 |
| cg10929784 | ROBO1 | 3 | 79773602 | 5'UTR | opensea | 2.90E-04 | 9.25E-02 | 0.663 | 0.643 | -0.020 |
| cg24315876 | RPTOR | 17 | 78913111 | Body | shelf | 2.66E-04 | 9.01E-02 | 0.855 | 0.881 | 0.026 |
| cg08328225 | RREB1 | 6 | 7183048 | Body | opensea | 1.32E-04 | 7.31E-02 | 0.656 | 0.631 | -0.025 |
| cg10752745 | SCHIP1 | 3 | 158991106 | 5'UTR | opensea | 1.69E-04 | 8.02E-02 | 0.438 | 0.477 | 0.040 |
| cg09108429 | SDCCAG8 | 1 | 243451540 | Body | opensea | 3.52E-04 | 9.81E-02 | 0.549 | 0.528 | -0.021 |
| cg25927227 | SFRP1 | 8 | 41127218 | Body | opensea | 3.25E-04 | 9.57E-02 | 0.590 | 0.567 | -0.022 |
| cg08572336 | SHANK1 | 19 | 51165404 | Body | island | 6.56E-05 | 6.00E-02 | 0.818 | 0.789 | -0.028 |
| cg18528054 | SLC22A20 | 11 | 64982895 | Body | opensea | 2.62E-04 | 8.98E-02 | 0.422 | 0.397 | -0.025 |
| cg15298607 | SLC25A25 | 9 | 130865618 | Body | opensea | 3.12E-05 | 4.82E-02 | 0.678 | 0.658 | -0.020 |
| cg02272859 | SLC34A2 | 4 | 25656514 | TSS1500 | shore | 3.19E-05 | 4.86E-02 | 0.679 | 0.652 | -0.028 |
| cg23750338 | SLC45A4 | 8 | 142222470 | Body | shore | 2.47E-04 | 8.87E-02 | 0.753 | 0.730 | -0.023 |
| cg02591213 | SLC5A11 | 16 | 24857208 | TSS1500 | opensea | 7.93E-06 | 3.65E-02 | 0.417 | 0.388 | -0.029 |
| cg20717474 | SLC5A11 | 16 | 24857188 | TSS1500 | opensea | 1.21E-05 | 3.77E-02 | 0.526 | 0.502 | -0.024 |
| cg07099998 | SLC5A11 | 16 | 24856891 | TSS1500 | opensea | 2.42E-06 | 2.46E-02 | 0.655 | 0.633 | -0.023 |
| cg01829163 | SLC7A5 | 16 | 87871160 | Body | opensea | 2.21E-05 | 4.49E-02 | 0.804 | 0.774 | -0.031 |
| cg11117131 | SLN | 11 | 107582818 | TSS200 | opensea | 6.32E-05 | 5.94E-02 | 0.758 | 0.781 | 0.022 |
| cg17567562 | SMARCC1 | 3 | 47687980 | Body | opensea | 2.17E-04 | 8.55E-02 | 0.566 | 0.533 | -0.033 |
| cg21870668 | SNRPN | 15 | 25123731 | 5'UTR | shore | 3.92E-05 | 5.19E-02 | 0.502 | 0.474 | -0.028 |
| cg23999078 | SNX9 | 6 | 158314486 | Body | opensea | 1.68E-04 | 8.01E-02 | 0.744 | 0.768 | 0.024 |
| cg14578284 | SOHLH1 | 9 | 138592347 | TSS1500 | shore | 5.84E-06 | 3.54E-02 | 0.827 | 0.805 | -0.021 |
| cg10534788 | SORCS2 | 4 | 7245919 | Body | opensea | 6.35E-05 | 5.94E-02 | 0.635 | 0.655 | 0.020 |
| cg03819945 | SPAG16 | 2 | 215243717 | Body | opensea | 3.61E-04 | 9.91E-02 | 0.626 | 0.650 | 0.024 |
| cg10574494 | SPATA18 | 4 | 52918457 | Body | shore | 3.64E-04 | 9.93E-02 | 0.221 | 0.194 | -0.027 |
| cg15706621 | SPTBN1 | 2 | 54861447 | Body | opensea | 1.85E-04 | 8.18E-02 | 0.630 | 0.653 | 0.023 |
| cg24259228 | SSC5D | 19 | 55999593 | TSS1500 | shore | 1.95E-04 | 8.29E-02 | 0.537 | 0.512 | -0.025 |
| cg20881311 | STK38L | 12 | 27457989 | Body | opensea | 2.57E-05 | 4.59E-02 | 0.667 | 0.637 | -0.030 |
| cg16096646 | SYNPO2 | 4 | 119771931 | 5'UTR | opensea | 2.95E-04 | 9.32E-02 | 0.361 | 0.406 | 0.045 |
| cg11368628 | SYT8 | 11 | 1856183 | Body | opensea | 8.21E-05 | 6.37E-02 | 0.402 | 0.379 | -0.023 |
| cg12958046 | TBC1D1 | 4 | 38019409 | Body | shelf | 9.36E-06 | 3.65E-02 | 0.691 | 0.656 | -0.035 |
| cg19437917 | TBCD | 17 | 80865872 | Body | shelf | 2.68E-04 | 9.03E-02 | 0.794 | 0.816 | 0.023 |
| cg03563169 | TBX18 | 6 | 85445250 | 3'UTR | opensea | 8.77E-05 | 6.48E-02 | 0.801 | 0.779 | -0.021 |
| cg05555876 | TCEA1 | 8 | 54935915 | TSS1500 | shore | 9.62E-06 | 3.65E-02 | 0.688 | 0.659 | -0.028 |
| cg19933954 | TCFL5 | 20 | 61494242 | TSS1500 | shore | 2.79E-04 | 9.14E-02 | 0.575 | 0.547 | -0.028 |
| cg05591105 | TENM4 | 11 | 78509989 | Body | opensea | 1.36E-05 | 3.88E-02 | 0.526 | 0.497 | -0.029 |
| cg13183732 | TMEM132D | 12 | 130091846 | Body | opensea | 1.78E-04 | 8.13E-02 | 0.697 | 0.718 | 0.021 |
| cg03992323 | TP53BP2 | 1 | 224023297 | Body | opensea | 2.88E-04 | 9.23E-02 | 0.414 | 0.380 | -0.034 |
| cg12268562 | TP73 | 1 | 3625409 | Body | shore | 1.36E-04 | 7.35E-02 | 0.481 | 0.455 | -0.026 |
| cg16407924 | TPO | 2 | 1452260 | Body | opensea | 8.78E-06 | 3.65E-02 | 0.651 | 0.626 | -0.024 |
| cg14931486 | TRAPPC4 | 11 | 118892305 | Body | shelf | 1.79E-04 | 8.13E-02 | 0.541 | 0.515 | -0.026 |
| cg05958922 | TRIM67 | 1 | 231319825 | Body | opensea | 2.84E-04 | 9.18E-02 | 0.532 | 0.558 | 0.026 |
| cg12547959 | TRIO | 5 | 14326153 | Body | opensea | 6.28E-05 | 5.94E-02 | 0.444 | 0.408 | -0.036 |
| cg10956093 | TRPM1 | 15 | 31341548 | Body | opensea | 2.90E-04 | 9.25E-02 | 0.368 | 0.331 | -0.036 |
| cg07875873 | TSKU | 11 | 76493379 | TSS1500 | shore | 8.90E-05 | 6.50E-02 | 0.576 | 0.552 | -0.023 |
| cg12958315 | TTBK1 | 6 | 43233455 | Body | shelf | 2.87E-04 | 9.21E-02 | 0.510 | 0.488 | -0.022 |
| cg11321181 | UNC13A | 19 | 17721489 | Body | shelf | 1.22E-04 | 7.21E-02 | 0.381 | 0.337 | -0.044 |
| cg11970806 | VEZF1 | 17 | 56066485 | TSS1500 | shore | 7.94E-05 | 6.36E-02 | 0.672 | 0.648 | -0.025 |
| cg19317830 | VGLL4 | 3 | 11675660 | Body | opensea | 3.03E-05 | 4.79E-02 | 0.212 | 0.184 | -0.027 |
| cg22707675 | WDR43 | 2 | 29116967 | TSS1500 | shore | 7.38E-05 | 6.18E-02 | 0.547 | 0.575 | 0.028 |
| cg04276715 | WDR46 | 6 | 33254460 | Body | shelf | 1.09E-05 | 3.67E-02 | 0.702 | 0.678 | -0.024 |
| cg16020118 | WDR88 | 19 | 33622600 | TSS1500 | shore | 2.54E-04 | 8.89E-02 | 0.620 | 0.599 | -0.022 |
| cg20341251 | WNT5B | 12 | 1754156 | Body | shore | 2.83E-05 | 4.71E-02 | 0.756 | 0.720 | -0.036 |
| cg03995300 | ZNF232 | 17 | 5019989 | 5'UTR | shore | 2.84E-04 | 9.18E-02 | 0.387 | 0.339 | -0.048 |
| cg00601727 | ZNF783 | 7 | 148989733 | Body | shore | 7.91E-05 | 6.36E-02 | 0.704 | 0.680 | -0.024 |

**Supplementary Table 1:** Abbreviations and definitions of the table are listed below:

CpG probe = probe ID/unique identified of the CpG site as per the Illumina CG database.

Gene = target gene name(s) as per the UCSC genome database.

Chr = chromosome containing the CpG, Bp = base pairs; the CpG’s location on the chromosome (GrCh37/hg19).

Feature = the type of gene region where the CpG is located

TSS200 = 0–200 bp upstream of the TSS, the transcriptional start site.

TSS1500 = 200–1500 bp upstream of the TSS.

5'UTR = 5' untranslated region, between the TSS and the start codon.

Body = Gene body; defined as between the start and stop codon (regardless of introns, exons, TSS, or promoter regions).

ExonBnd = boundary between an exon and an intron.

3'UTR = 3' untranslated region, between the stop codon and the poly A tail.

Cgi: denotes the location of a particular CpG relative to the closest CpG island.

Island: located within the CpG island.

Shore: 0-2kb from the CpG island.

Shelf: 2-4kb from the CpG island.

Opensea: Isolated CpGs in the genome

**Supplementary Table 2: Significant DMPs with Δ β ≥±2% identified between NRES and RES located in differentially expressed gene regions.**

| **Probe ID** | **Gene** | **CHR** | **Location** | **Feature** | **Cgi** | **p value** | **FDR (q)** | **Δ β** |
| --- | --- | --- | --- | --- | --- | --- | --- | --- |
| cg02745111 | ATMIN | 16 | 81070647 | Body | shore | 9.60E-05 | 0.07 | -0.02 |
| cg13104274 | ATP1B1 | 1 | 169078316 | Body | shore | 4.77E-05 | 0.06 | -0.02 |
| cg26009832 | ATP1B1 | 1 | 169081894 | Body | opensea | 9.50E-05 | 0.07 | -0.02 |
| cg19677267 | CD52 | 1 | 26645161 | Body | opensea | 2.10E-04 | 0.08 | 0.03 |
| cg12001491 | CD52 | 1 | 26645487 | Body | opensea | 3.50E-04 | 0.10 | 0.04 |
| cg23687322 | CHN2 | 7 | 29523056 | TSS1500 | opensea | 1.93E-04 | 0.08 | -0.05 |
| cg06926818 | CHN2 | 7 | 29523160 | TSS1500 | opensea | 9.67E-05 | 0.07 | -0.04 |
| cg06279274 | FAM24B | 10 | 124635805 | 5'UTR | shelf | 1.31E-04 | 0.07 | -0.03 |
| cg06705237 | FBP1 | 9 | 97402555 | TSS200 | shore | 2.47E-04 | 0.09 | -0.02 |
| cg14422240 | FTSJD2 | 6 | 37425031 | Body | opensea | 7.06E-05 | 0.06 | -0.03 |
| cg08584037 | JAK2 | 9 | 4984071 | TSS1500 | shore | 3.14E-04 | 0.09 | -0.02 |
| cg22200736 | KRT72 | 12 | 52995358 | TSS200 | shore | 3.20E-04 | 0.10 | -0.02 |
| cg04453169 | MAP7 | 6 | 136680760 | Body | shore | 3.94E-05 | 0.05 | -0.04 |
| cg18581616 | NLRP8 | 19 | 56478019 | Body | opensea | 4.25E-06 | 0.03 | 0.03 |
| cg03485252 | NRG1 | 8 | 31503975 | Body | opensea | 1.05E-05 | 0.04 | 0.02 |
| cg21870668 | SNRPN | 15 | 25123731 | 5'UTR | shore | 3.92E-05 | 0.05 | -0.03 |
| cg22707675 | WDR43 | 2 | 29116967 | TSS1500 | shore | 7.38E-05 | 0.06 | 0.03 |

**Supplementary Table 2:** NRES = average NRES β values at a CpG site. RES = average RES β values at a CpG site. Δ β = change in methylation relative to responders. For a detailed description of other terms in the table, please see Supplementary Table 1.

**Supplementary Table 3: Validation and Replication results at CpG probes in *CHN2* and *JAK2*.**

|  | | **Validation** | | **Replication** | |
| --- | --- | --- | --- | --- | --- |
| **Probe ID** | **Gene** | **p value** | **ΔBeta** | **p value** | **ΔBeta** |
| **cg23687322** | CHN2 | 4.33E-04 | -0.05 | 0.17 | -0.03 |
| **cg06926818** | CHN2 | 1.43E-03 | -0.04 | 0.03 | -0.03 |
| **cg08584037** | JAK2 | 9.47E-04 | -0.02 | 0.59 | -0.003 |

**Supplementary Table 3:** Directional change in methylation values (∆Beta) of cg23687322, cg06926818, and cg08584037 probes relative to responders, along with significance levels.

**Supplementary Table 4: Blood cell count comparison between NRES, RES and HC**.

|  | **% Lymphocytes** | **% Monocytes** | **% Neutrophils** | **% Eosinophils** | **% Basophils** |
| --- | --- | --- | --- | --- | --- |
| **HC** | 33.50 ± 7.55 | 8.05 ± 2.13 | 55.14 ± 9.12 | 2.72 ± 2.04 | 0.48 ± 0.64 |
| **NRES** | 31.18 ± 7.62 | 7.87 ± 2.38 | 57.60 ± 9.18 | 2.69 ± 3.50 | 0.43 ± 0.64 |
| **RES** | 31.39 ± 8.29 | 7.60 ± 2.05 | 58.21 ± 9.27 | 2.28 ± 1.90 | 0.40 ± 0.51 |
| **F value** | 2.22 | 0.82 | 2.53 | 0.7 | 0.36 |
| **P value** | 0.11 | 0.44 | 0.081 | 0.5 | 0.7 |

**Supplementary Table 4:** Cell type % averages are shown for all 3 groups, followed by standard deviation values. NRES = non-responder, RES = responder, HC = healthy controls.

**Supplementary Methods**

EPIC microarray data processing and differential methylation analysis.

Raw .IDAT files were received from Genome Quebec (GQ) for the discovery cohort, and Illumina for the discovery and replication cohorts respectively after an initial quality control assessment of methylation detection using GenomeStudio software (Illumina, USA). Ratios of the median unmethylated and methylated intensities were used to perform an additional sample quality control (QC) measurement in R (ver 3.4). Samples were removed if either channel intensity fell below 10.5. Further bioinformatics processes were conducted in house using the Chip Analysis Methylation Pipeline (*ChAMP*) Bioconductor package. This user-friendly pipeline was designed for the purpose of analysing Illumina 450K Beadchip data, but has since been updated for the more comprehensive EPIC Beadchip array (1). This pipeline was chosen given that it provides a straightforward workflow that incorporates multiple different Bioconductor packages to perform various steps of microarray processing. A majority of the algorithms are based on the *minfi* Bioconductor package (2). Raw intensity data files were used to load the data into the R environment with the *champ.load* function, which also allows for probe QC and removal steps to occur simultaneously. Probes with low detected signals (n=10,164), cross reactive probes (n= 44), non-CpG probes (n=2,913**), probes with less than three beads in at least five percent of samples per probe (n= 170),** probes that bound to SNP sites (n=150,294; removed as per Zhou et al’s recommendations (3) and additionally removed any probes that targeted known SNP sites, as this affects probe hybridization accuracy), and sex chromosome probes (n=17,245) are all considered problematic for accurate downstream methylation detection. After removing these probes, 679, 362 probes remained for downstream analysis. Although this may seem like a drastic decrease in the number of retained probes, this is normal during microarray pre-processing steps (4) (5). For each CpG, beta values were calculated as the methylation signal over the sum of unmethylated and methylated signals, analogous to the percent of methylation at each CpG site. Beta values were then normalized using the *champ.norm* function, specifically with the beta mixture quartile method (BMIQ function). Beta distribution graphs were analyzed to ensure that most probes fell within canonical 0-0.2 and 0.8-1 ranges suggestive of unmethylated or methylated levels, respectively, and normalized with a beta-mixture quantile normalisation (BMIQ). BMIQ is an intra-sample normalization method that adjusts for the type 1 and type 2 bead hybridization differences on the EPIC microarray. It transforms the type 2 probe probabilities into quantiles of type 1 probe distributions, while performing a conformal transformation of hemimethylated probes given that they do not fall under standard beta distributions (6). Next, the singular value decomposition (SVD) method was called by *champ.SVD* in order to assess the amount and significance of technical batch components, along with any potential confounding variables, in our dataset. Using the *champ.runCombat* function, Combat algorithms were applied in order to correct for our two initial submission batches, along with slide and array as technical batch components detected by SVD. Combat relies on parametric empirical Bayes frameworks when adjusting data for batch effects in a manner suitable for larger sample sizes (7).

Age and sex were corrected for as covariates after being identified as confounding biological components through SVD. Differentially methylated positions (DMPs) were identified using the function *champ.DMP* (p = 0.05, q= 0.1)(8). M values (log_2_ transformed beta values) were used for all analyses to avoid heteroscedasticity as recommended by Du et al (9).

Genome wide mRNA gene expression analysis on the HT-12 Beadchip

Whole blood was collected in EDTA tubes containing LeukoLOCK filters (Thermo Scientific, USA) from healthy controls and depressed patients. LeukoLOCK filters isolate leukocytes from whole blood, and eliminate the interference of globin mRNA from red blood cells (RBCs) while conducting expression analyses (10). RNA was extracted as per a modified LeukoLOCK Total RNA Isolation protocol.  Agilent 2200 Tapestation was used to assess RNA quality across samples, with a RNA integrity number (RIN) cutoff of six, and sent to GQ in two batches. There, further RNA QC was conducted, complementary RNA was prepared using standard Illumina Whole-Genome protocols, and was hybridized to Human HT-12 v4 Expression BeadChips (Illumina, USA). Differential gene expression analysis was conducted on the Human HT-12 v4 Expression Beadchip (Illumina, USA), which provides accurate genome wide expression coverage on up to 47,000 well-known genes, gene candidates, and splice variants. Initial quality control of raw probe signals was conducted in GenomeStudio by GQ.

HT-12 Expression Beadchip analysis

Only the subset of samples that appeared in our DNA methylation analysis were included for whole genome expression analysis. Raw probe intensities were loaded into the R environment with the *limma* Bioconductor package, a commonly used package for analyzing differential expression on microarrays (11). Probe signals were detected with the *propexp* function, and normalized with the *normalizeBetweenArrays* function. Probe filtering was conducted using a detection p value of < 0.01 in at least 20% of samples cut off to denote retained probes, where 16,378 gene probes were preserved for downstream analysis. Differentially expressed genes were identified through linear regression analyses, with age and sex as covariates. All analyses were conducted with log_2_ transformed values. Only probes with at least ±0.1 logFC values were included in order to only include the most biologically relevant genes.

Targeted bisulfite sequencing

*Primer Design*

Bisulfite sequencing primers were designed for our target amplicons with the Methyl Primer Express software (ThermoFisher Scientific). Primer sequences for probes in *CHN2* and *JAK2* are specified below:

*CHN2 (*cg23687322 and cg06926818)

- Forward sequence: ATTTTAGAGAGGAGTTTGTTAATTTTAT
- Reverse sequence: ACTTCTCAAACAAAACTTATCTAAAC

*JAK2* (cg08584037 and cg08339825)

- Forward sequence: GTATTTTGATGGAAGYGATAAAATAATA
- Reverse sequence: TAAAATTCTTTTCCCAAATAATCATAAAAC

When assessing CpGs in FAM24B genomic regions, a portion of a CpG island was located within a primer sequence. Thus, CpGs in forward primers were replaced with a 50% mix of C and T, while in reverse primers, they were replaced with a 50% mix of A and G to establish equal attachment between methylated and unmethylated templates as much as possible. The second round of primers were designed by adding CS1 and CS2 sequences to our forward and reverse targeted amplicon primers respectively:

CS1: 5’ ACACTGACGACATGGTTCTACNNN 3’

CS2: 5’ TACGGTAGCAGAGACTTGGTCTNNN 3’

3 N bases (25% mix of all 4 bases) were added between our targeted primers to increase base diversity and improve sequencing QC in early PCR amplification cycles. Our third and final round of primers were designed to target CS1 and CS2 sequences by attaching P5 and P7 Illumina flow-cell attachment sequences to our CS1 and CS2 primers:

P5: 5' AAT GAT ACG GCG ACC ACC GA 3'

P7: 5' CAA GCA GAA GAC GGC ATA CGA GAT 3'

The forward primer was designed to amplify CS1-amplicon regions, and attach the P5 flow cell clustering sequence. The reverse primer was designed to amplify CS2-amplicon regions followed by addition of a Fluidigm indexing barcode for sample identification and the P7 flow cell clustering sequence. After testing our initial amplicon primers in bisulfite converted DNA from our peripheral blood based samples, we also tested them in bisulfite converted DNA collected from post mortem brain samples to confirm that our genes are expressed in brain and blood.

Amplicon library preparations and loading samples onto the MiSeq

Three rounds of multiplexed PCR amplification were performed using 384 well plates to prevent intra-amplicon batch effects. Using freshly bisulfite converted DNA, the first round of PCR amplification was completed using primers targeting our amplicons. The second round of PCR was performed using with first round PCR products and amplicon primers with CS1 and CS2 sequences attached. The final round of PCR reaction was performed with primers that attach P5 and P7 Illumina flow-cell binding sequences along with unique indexing barcodes per sample. After each round of amplification, a small amount of all samples was run on a two percent agarose gel to ensure all samples were amplified correctly, followed by purification with Agencourt AMPure XP (AMPure) beads (Beckman Coulter, Cat. #: A63881). KAPA HiFi HotStart Uracil+ (Kapa Biosystems, Cat #: KK2802) was used in only the first amplification round, followed by KAPA HiFi HotStart ReadyMix (2X) (Kapa Biosystems, Cat #: KK2602) in rounds two and three. To quantify our final amplicon library’s concentrations, three different methods were used (Agilent 2200 TapeStation, NanoDrop ND-1000 and qPCR) prior to MiSeq loading to ensure that our pooled samples had an optimal final molarity of 2nM. Samples were loaded onto the MiSeq platform with the V3 600 cycle kit (Illumina, Cat #: MS-102-3003).

**References:**

1. Morris TJ et al. ChAMP: 450k Chip Analysis Methylation Pipeline. Bioinformatics. 2014;30(3):428-30.

2. Aryee MJ et al. Minfi: a flexible and comprehensive Bioconductor package for the analysis of Infinium DNA methylation microarrays. Bioinformatics. 2014;30(10):1363-9.

3. Zhou W, Laird PW, Shen H. Comprehensive characterization, annotation and innovative use of Infinium DNA methylation BeadChip probes. Nucleic Acids Research. 2017;45(4):e22-e.

4. Houtepen LC et al. Genome-wide DNA methylation levels and altered cortisol stress reactivity following childhood trauma in humans. Nature Communications. 2016;7:10967.

5. Kuan PF et al. An epigenome-wide DNA methylation study of PTSD and depression in World Trade Center responders. Translational Psychiatry. 2017;7:e1158.

6. Teschendorff AE et al. A beta-mixture quantile normalization method for correcting probe design bias in Illumina Infinium 450k DNA methylation data. Bioinformatics. 2013;29(2):189-96.

7. Johnson W, Rabinovic, A, and Li, C. Adjusting batch effects in microarray expression data using Empirical Bayes methods. . Biostatistics 2007;8(1):(1):118-27.

8. Ritchie ME PB et al. “limma powers differential expression analyses for RNA-sequencing and microarray studies.”. Nucleic Acids Research. 2015;43(7), pp. e47.

9. Du P et al. Comparison of Beta-value and M-value methods for quantifying methylation levels by microarray analysis. BMC Bioinformatics. 2010;11:587-.

10. Schwochow D, Serieys LEK, Wayne RK, Thalmann O. Efficient recovery of whole blood RNA - a comparison of commercial RNA extraction protocols for high-throughput applications in wildlife species. BMC Biotechnology. 2012;12:33-.

11. Ritchie ME PB et al. “limma powers differential expression analyses for RNA-sequencing and microarray studies.” Nucleic Acids Research. 2015;43(7), pp. e47.

12. Chen GG et al. Medium throughput bisulfite sequencing for accurate detection of 5-methylcytosine and 5-hydroxymethylcytosine. BMC Genomics. 2017;18:96.
