# Supplementary figures and images for: Integrated genome-wide methylation and expression analyses reveal functional predictors of response to antidepressants
Source: Transl Psychiatry. 2019 Oct 8;9:254. doi: 10.1038/s41398-019-0589-0 (PMC6783543; doi:10.1038/s41398-019-0589-0)

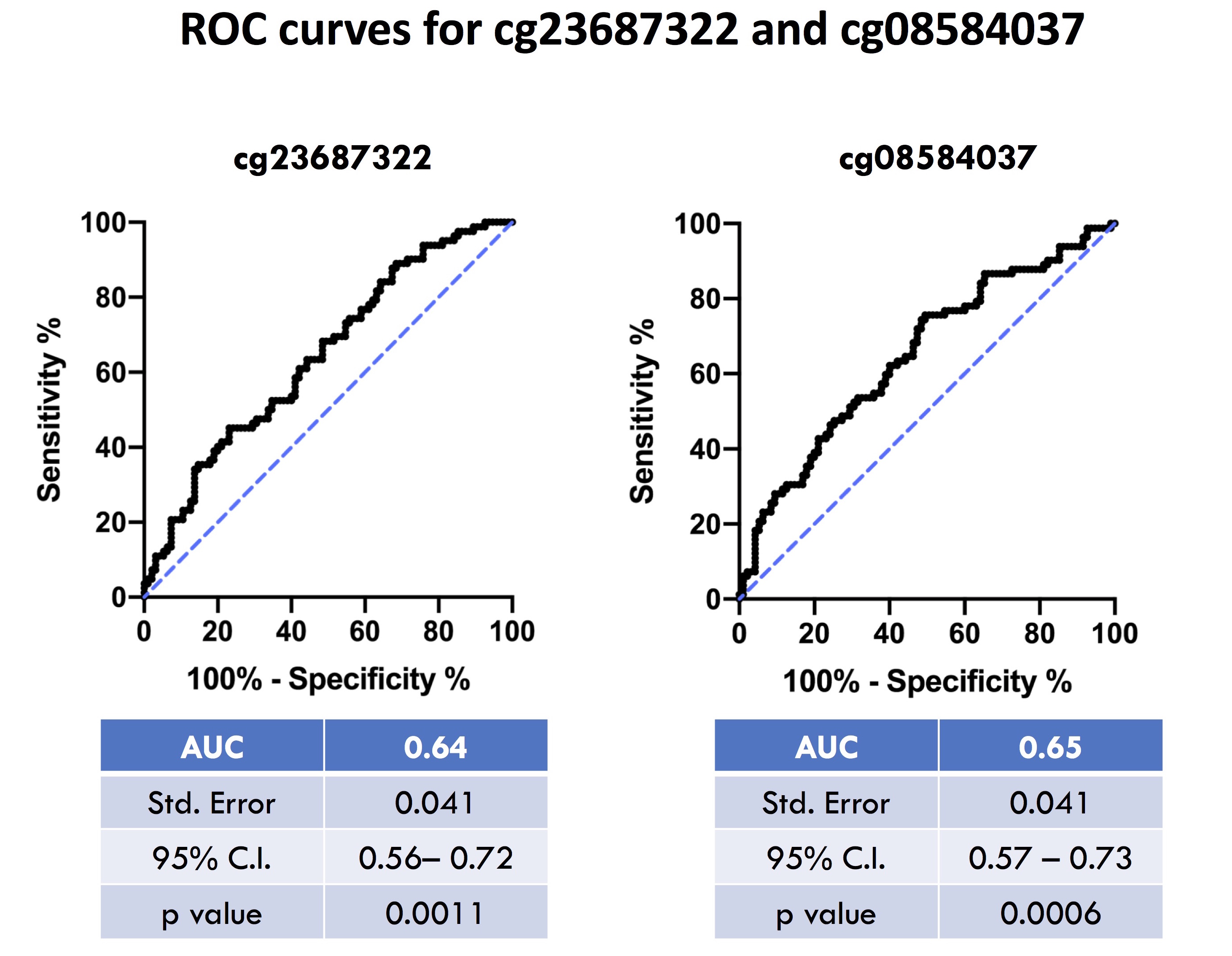

Supplement: Supplementary file 2 — Supplementary Figure 1 [file 41398_2019_589_MOESM2_ESM.jpg]
