# Supplementary material for: Integrated genome-wide methylation and expression analyses reveal functional predictors of response to antidepressants
Source: Transl Psychiatry. 2019 Oct 8;9:254. doi: 10.1038/s41398-019-0589-0 (PMC6783543; doi:10.1038/s41398-019-0589-0)
Supplement: Supplementary file 3 — Supplementary Figure 1 Legend [file 41398_2019_589_MOESM3_ESM.docx]

**Supplementary Figure 1:** ROC curves for cg23687322 and cg08584037**,** derived from methylation values of our discovery cohort. AUC = Area under the curve. C.I. = Confidence Interval.
